# Supplementary material for: Gene expression correlates of social evolution in coral reef butterflyfishes
Source: Proc Biol Sci. 2020 Jun 24;287(1929):20200239. doi: 10.1098/rspb.2020.0239 (PMC7329040; doi:10.1098/rspb.2020.0239)
Supplement: Supplementary methods and tables [file rspb20200239supp1.pdf]

Gene expression correlates of social evolution in coral reef butterflyfishes: Supplementary materials

Jessica P. Nowicki[1,2], Morgan S. Pratchett[1], Stefan P. W. Walker[1], Darren J. Coker[1,3], Lauren A. O'Connell[2]*

[1]ARC Centre of Excellence for Coral Reef Studies, James Cook University, Townsville, QLD 4810, Australia

[2]Department of Biology, Stanford University, Stanford, CA, 94305, USA

[3]Red Sea Research Center, Division of Biological and Environmental Science and Engineering, King Abdullah University of Science and Technology, Thuwal, 23955-6900, Saudi Arabia

*To whom correspondence should be addressed:

Lauren O'Connell

Department of Biology

Stanford University

371 Jane Stanford Way

Stanford, CA 94305

loconnel@stanford.edu

Journal: Proceedings of the Royal Society, B

DOI: 10.1098/rspb.2020.0239

Supplementary Methods S1: Detailed qPCR gene expression analysis

Frozen brains were coronally sectioned on a cryostat at 100µm, thaw mounted onto Superfrost Plus slides (Fisher Scientific) and stored at -80˚C. Brain regions were identified using a butterflyfish brain atlas (Bauchot, 1989; Dewan and Tricas, 2014) and manually extracted at -30˚C using a hand-held micro-punching device (50mm diameter; Stoelting, model # 57401) (**Fig. 2 inset**). Punches from individual brain regions were pooled in RNA*later*™ (ThermoFischer, cat no. AM7020) at 4˚C overnight, and then stored at -20˚C for up to one week. Tissue punches were transferred into a lysis buffer and homogenized by passing through a 21-gauge needle 15 times. RNA was extracted using an E.Z.N.A® HP Total RNA kit (Omega Bio Tek, cat no # R6812-02) according to the manufacturer's instructions, and stored at -80˚C prior to cDNA synthesis. RNA was reverse transcribed into cDNA using Superscript III reverse transcriptase (Life Technologies) and gene-specific primers (see Table S1 for primer sequences). Residual primers and salts from reverse transcription were removed using an E.Z.N.A ® Tissue DNA purification kit (Omega, product # D3396-02), and cDNA was stored at -20˚C for up to 10 days prior to qPCR.

The whole brain transcriptome of *C. lunulatus* was sequenced as a reference for designing species specific cloning primers for target genes. One *C. lunulatus* brain was taken out of RNAlater, rinsed in 1X phosphate buffered saline (PBS) and placed immediately in Trizol (Life Technologies, Grand Island, NY) where RNA was extracted according to manufacturer instructions. Poly-adenylated RNA was isolated from each sample using the NEXTflex PolyA Bead kit (Bioo Scientific, Austin, TX, USA). Lack of contaminating ribosomal RNA was confirmed using the Agilent 2100 Bioanalyzer. A strand specific library was prepared using the dUTP NEXTflex RNAseq kit (Bioo Scientific), which includes a magnetic bead-based size selection of roughly 350 bp. The library was pooled in equimolar amounts with samples from an unrelated study after library quantification using both quantitative PCR with the KAPA Library Quantification Kit (KAPA Biosystems, Wilmington, MA, USA) and the fluorometric Qubit dsDNA high sensitivity assay kit (Life Technologies), both according to manufacturer instructions. Libraries were sequenced on an Illumina HiSeq 2000 to obtain paired-end 100bp reads. We first corrected errors in the Illumina reads using Rcorrector (parameters: run_rcorrector.pl -k 31) and then applied quality and adaptor trimming using Trim Galore! (http://www.bioinformatics.babraham.ac.uk/projects/trim_galore/; parameters: trim_galore --paired --phred33 --length 36 -q 5 --stringency 5 --illumina -e 0.1). After filtering and trimming, a total of 64,795,096 paired reads remained for de novo assembly. We created a *C. lunulatus de novo* transcriptome assembly using Trinity (parameters: --seqType fq --SS_lib_type RF). The raw Trinity assembly produced 376,338 contigs (N50: 1148 bp). Raw data and the *C. lunulatus* transcriptome is available on read archive (submission pending acceptance).

Using the *C. lunulatus* transcriptome, primers were designed to clone target gene sequences. Sequences of *OTR*, *V1aR*, *D1R*, *D2R* and *MOR* genes were then used to design qPCR primers that flanked exon boundaries using *Danio rerio* and *Stegastes partitus* genomes as a reference (see Table S1). Prior to qPCR, primer sets and instrument cycling parameters were empirically optimized on standard curves using

several metrics of quality control (i.e., assay amplification R^2 value of at least 0.95, assay slope of approximately -3.3, assay melting curve that only produced a single amplicon peak, no amplicon signal in the no template control (NTC) or no reverse-transcriptase control (NRTC)). Quantitative PCR was then performed on each sample using a reaction mixture and qPCR cycling instrument (CFX380) recommended by the enzyme manufacturer (see Table S2 for parameters). Samples were run in technical triplicate on 384 well qPCR plates with standard curves in order to determine assay efficiency. Not all regions of each brain were measured for gene expression due to insufficient tissue available.

References:

1. Bauchot R, Ridet JM, Bauchot ML. 1989 The brain organization of butterflyfishes. *Environ. Biol. Fishes* **25**, 204–219.

2. Dewan AK, Tricas TC. 2014 Cytoarchitecture of the telencephalon in the coral reef multiband butterflyfish (*Chaetodon multicinctus*: Perciformes). *Brain Behav. Evol.* **84**, 31–50.

Supplementary Methods S2: R script for analysis of qPCR data

<u>*Chaetodon lunulatus*: *Social system X sex:*</u>

```
# 1. install.packages("MCMC.qpcr")

library(MCMC.qpcr)


# 2. read in data

data <- read.csv(file="Nowicki_C_Lun_M&F_data_ESM.csv")

data$count <- as.numeric(data$count) # force counts to be numeric

head(data) # allows you to view your data to ensure it was imported correctly


# 3. sub-set data into different brain regions and store them in separate data frames, in order to analyse each brain region separately

levels(data$region_mammal) #shows names of brain region levels

levels(data$region_mammal) #shows names of brain region levels

Hipp=subset(data, region_mammal=="Hipp") #makes a dataframe for Hipp

blAMY=subset(data, region_mammal=="blAMY")

LS=subset(data, region_mammal=="LS")

meAMY.BNST=subset(data, region_mammal=="meAMY.BNST")

NAcc=subset(data, region_mammal=="NAcc")

POA=subset(data, region_mammal=="POA")

Str.CP=subset(data, region_mammal=="Str.CP")

VTA=subset(data, region_mammal=="VTA")
```

Do remaining steps for each brain region data frame separately. Here is the example using blAMY

```
# 4. model fitting

table(blAMY$sex, blAMY$social) # shows you table of all combinations of all factos, for N reporting purposes

## specify your "reference" comparisons

blAMY$sex = relevel(blAMY$sex, ref='M')
```

blAMY$social = relevel(blAMY$social, ref='PB')

fit naive model (assumes no control genes)

blAMY_naive_model=mcmc.qpcr(data=blAMY, fixed="sex+social+sex:social",random = c("individual"), nitt=510000,thin=500,burnin=10000)

summary(blAMY_naive_model) # this shows results from fully-crossed 2-way fixed effects

HPDsumm_blAMY_naive=HPDsummary(blAMY_naive_model, blAMY, relative=T) #plot with HDPsummary to visualize whether there are global effects (i.e., all genes appear to be up- or down-regulated under some conditions

5. decision time

if no global effects are present in naive model, try sharpening credible intervals by running an "informed" model instead, but only if your control genes are reasonably stable in the naive model (as per HPDsymmary plot).

if global effects ARE present in naive model, then fit a "soft normalization" model (pg. 30 Matz tutorial, step 10)

testing whether informed model sharpens credible intervals more than naive model:

blAMY_naive_model = mcmc.qpcr(data=blAMY, fixed="sex+social+sex:social", random = "individual",nitt=510000,thin=500,burnin=10000, controls=c("r18S"), include=0)

blAMY_informed_model = mcmc.qpcr(data=blAMY, fixed="sex+social+sex:social", random = "individual", controls=c("r18S"), m.fix=1.2, nitt=510000,thin=500,burnin=10000)

HPDplot(model=blAMY_naive_model, factors="socialSol", main="social_system", hpdtype="l")

HPDpoints(model=blAMY_informed_model, factors="socialSol", hpdtype="l", col="coral") #plots credible interval difference between naive and informed

6. In the best fit model, conduct pair-wise comparisons of p-values:

blAMY_naive_pwp = HPDsummary(model=blAMY_naive_model, data=blAMY) # shows absolute abundances

blAMY_naive_pwp$summary # retrieves bundles of data that can be used for more plotting (means, sds, CIs)

blAMY_naive_pwp$geneWise # calculates pairwise comparisons

7. plot results

plot_blAMY_naive=HPDsummary(blAMY_naive_model, blAMY,xgroup="social") # trellis plot of all genes together in one panel

trellisByGene(plot_blAMY_naive, xFactor="social", groupFactor = "sex")+xlab("social system") # trellis plot of genes shown in separate panels

#end

Chaetodon species: Social system X sex

1. install.packages("MCMC.qpcr")

library(MCMC.qpcr)

2. read in data

data <- read.csv(file="Nowicki_C_SPP_M&F_data_ESM.csv")

data$count <- as.numeric(data$count) # force counts to be numeric

head(data) #allows you to view your data to ensure it was imported correctly

3. sub-set your data into different brain regions and store them in separate data frames, in order to analyze each brain region separately

levels(data$region_mammal) #shows names of brain region levels

Hipp=subset(data, region_mammal=="Hipp") #makes a dataframe for Hipp

blAMY=subset(data, region_mammal=="blAMY")

LS=subset(data, region_mammal=="LS")

meAMY.BNST=subset(data, region_mammal=="meAMY.BNST")

NAcc=subset(data, region_mammal=="NAcc")

POA=subset(data, region_mammal=="POA")

Str.CP=subset(data, region_mammal=="Str.CP")

VTA=subset(data, region_mammal=="VTA")

Do remaining steps for each brain region data frame separately. Here is the example using blAMY:

4. model fitting

```
table(blAMY$sex, blAMY$social) # shows you table of all combinations of all factos, for N reporting purposes

## specify your "reference" comparisons

blAMY$sex = relevel(blAMY$sex, ref='M') #sets M as reference for sex factor

blAMY$social = relevel(blAMY$social, ref='PB') #sets PB as reference for social factor

## fit naive model (assumes no control genes)

blAMY_naive_model=mcmc.qpcr(data=blAMY, fixed="sex+social+sex:social",random = c("individual"), nitt=510000,thin=500,burnin=10000)

summary(blAMY_naive_model) # this shows results from fully-crossed 2-way fixed effects

blAMY_HPDsumm_naive_model=HPDsummary(blAMY_naive_model, blAMY, relative=T) #plot with HDPsummary to visualize whether there are global effects (i.e., all genes appear to be up- or down-regulated under some conditions)


# 5. decision time

## if no global effects are present in naive model, try sharpening credible intervals by running an "informed" model instead, but only if your control genes are reasonably stable in the naive model (as per HPDsymmary plot).

## if global effects ARE present in naive model, then fit a "soft normalization" model (pg. 30 tutorial, step 10).

### testing whether informed model sharpens credible intervals more than naive model

blAMY_naive_model = mcmc.qpcr(data=blAMY, fixed="sex+social+sex:social", random = "individual",nitt=510000,thin=500,burnin=10000, controls=c("r18S"), include=0) #analyzes sex-specific effects of social system

blAMY_informed_model = mcmc.qpcr(data=blAMY, fixed="sex+social+sex:social", random = "individual", controls=c("r18S"), m.fix=1.2, nitt=510000,thin=500,burnin=10000) #analyzes sex-specific effects of social system

HPDplot(model=blAMY_naive_model, factors="socialSol", main="social_system", hpdtype="l") #plots credible interval of naive model

HPDpoints(model=blAMY_informed_model, factors="socialSol", hpdtype="l", col="coral") #plots credible interval difference between naive and informed


# 6. in best fit model, conduct pair-wise comparisons of p-values

blAMY_informed_pwp = HPDsummary(model=blAMY_informed_model, data=blAMY) # shows absolute abundances
```

blAMY_informed_pwp$summary # retrieves bundles of data that can be used for more plotting (means, sds, CIs)

blAMY_informed_pwp$geneWise # calculates pairwise comparisons

7. plot results

plot_blAMY_infor=HPDsummary(blAMY_informed_model, blAMY,xgroup="social") # trellis plot of all genes together in same panel

trellisByGene(plot_blAMY_infor, xFactor="social", groupFactor = "sex")+xlab("social system") # trellis plot of each gene in its own panel

#end.

Chaetodon species: Males: OTR (ITR) in meAMY.BNST (Vs):

1. install.packages("MCMC.qpcr")

library(MCMC.qpcr)

2. read in data

data <- read.csv(file="Nowicki_C_SPP_Males_data_ESM.csv")

data$count <- as.numeric(data$count) # force counts to be numeric

head(data) # allows you to view your data to ensure it was imported correctly

levels(data$sex) # shows you all levels of the sex factor, to sanity check the data set looks correct

3. sub-set data into different brain regions and store them in separate data frames, in order to analyze each brain region separately

levels(data$region_mammal) #shows names of brain region levels

Hipp=subset(data, region_mammal=="Hipp") #makes a dataframe for Hipp

blAMY=subset(data, region_mammal=="blAMY")

LS=subset(data, region_mammal=="LS")

meAMY.BNST=subset(data, region_mammal=="meAMY.BNST")

NAcc=subset(data, region_mammal=="NAcc")

POA=subset(data, region_mammal=="POA")

Str.CP=subset(data, region_mammal=="Str.CP")

VTA=subset(data, region_mammal=="VTA")

4. model fitting

meAMY.BNST$species = relevel(meAMY.BNST $species, ref='C.bar') #sets C.bar as "reference" for species factor

meAMY.BNST_naive_model=mcmc.qpcr(data=meAMY.BNST, fixed="species", random = "individual", nitt=510000,thin=500,burnin=10000, pr=TRUE)

summary(meAMY.BNST_naive_model) # this shows results from 1-way fixed effects

meAMY.BNST_HPDsumm_naive_model=HPDsummary(meAMY.BNST_naive_model, meAMY.BNST, relative=T) #eyeballametrically determine whether there are global effects.

5. decision time

if no global effects are present in naive model, try sharpening credible inervals by running an "informed" model instead.

if global effects ARE present in naive model, then fit a "soft normalization" model (pg. 30 Matz tutorial, step 10)

testing whether informed model sharpens credible intervals more than naive model:

MeAMY.BNST_naive_model = mcmc.qpcr(data=MeAMY.BNST, fixed="species", random = "individual", controls=c("r18S"), include=0, nitt=510000,thin=500,burnin=10000) #runs naive model

MeAMY.BNST_informed_model = mcmc.qpcr(data=MeAMY.BNST, fixed="species", random = "individual", controls=c("r18S"), m.fix=1.2, nitt=510000,thin=500,burnin=10000) #runs informed model

HPDplot(model=MeAMY.BNST_naive_model, factors="speciesC.lun", main="C.lun", hpdtype="l") #plots credible interval of naive model

HPDpoints(model=MeAMY.BNST_informed_model, factors="speciesC.lun", hpdtype="l", col="coral") #plots credible interval difference between naive and informed

6. In the best fit model, conduct pair-wise comparisons of p-values

MeAMY.BNST_spp_informed_pwp = HPDsummary(model=MeAMY.BNST_informed_model, data=MeAMY.BNST) # shows absolute abundances

MeAMY.BNST_spp_informed_pwp$summary # retrieves bundles of data that can be used for more plotting (means, sds, CIs)

MeAMY.BNST_spp_informed_pwp$geneWise # calculates pairwise difference between treatments and their statistical significances; upper triangle is log fold changes, lower triangle is the corresponding p-values

```
# 7. plot results

spp_order<-c("C.vag", "C.lun", "C.bar", "C.trif", "C.rainf", "C.pleb")

plot_meAMY.BNST_informed=HPDsummary(meAMY.BNST_informed_model,
meAMY.BNST,xgroup="species", x.order=spp_order) # trellis plot of all genes in same panel

trellisByGene(plot_meAMY.BNST_informed, xFactor="species", groupFactor =
"species")+xlab("species") # trellis plots of genes in separate panels

#end.
```

Supplementary Table S1: Primer sequences used to reverse transcribe, clone, and preform quantitative qPCR on *Chaetodon* butterflyfish

Target gene	Cloning primers	Reverse Transcription primers	qPCR primers
ITR	Pair a (using *C.lunulatus* transcriptome):<br>F: 5'-TTTTGTGCAGGTTGGTGAAA<br>R:5'-AGATCCAGGGGTTACAGCAG<br><br>Pair b (to obtain exon sequence):<br>F: 5'- TTTTGTGCAGGTTGGTGAAA<br>R: 5'- GAATTGAACCGCTGGATGTT	R:5'- GGCTGCTCGTGCTTTTAATG	F:5'- GTCTGTTGGACCCCCTTTTT<br>R:5'- CAGCAGCATGGAGATGATGA
V1aR	Pair a (using *C.lunulatus* transcriptome):<br>F: 5'-GGAAGACGATGACTGGTGCT<br>R: 5'-AGCTGTTGAGACTGGCAAGG<br><br>Pair b (to obtain exon sequence):<br>F: 5'- GGAAGACGATGACTGGTGCT<br>R: 5'- TGTTAGACCTCCTGGCTGCT	R:5'-AGGGCTTCGATTGGTCATCT	F:5'-CTGTGTGGGATGAAAACTTCCT<br>R:5'-AGGAGGTGACCGCTGAAGAT
D1R	Pair a (using *C.lunulatus* transcriptome):<br>F: 5'-GAACGCAAGATGACCCCTAA<br>R: 5'-CCTGTCAGGCATGTCCTTTT	R:5'-TCAAAGGTGGAGCTGAT	F:5'-TCGAACATGGAGAGTGAGAGC<br>R:5-CCAGCAGCACACAAACACTC
D2R	Pair a (using *C.lunulatus* transcriptome):<br>F: 5'-TTGCTGTAAGCTGCCATTTG<br>R: 5'-TTTTGCCTGAAACAGGTCA<br><br>Pair b (using *C.lunulatus* transcriptome):<br>F: 5'-TCTGTGGTGTGGGTGCTGT<br>R: 5'-CACGCCTTGCAATAAGACAC	R:5'-TCTGTTGCAGGATCTCCATTC	F:5'-AACGGGAGCTTTCCTGTCA<br>R:5'-GCTGTTGTTCAGCTCATCCAG
MOR	Pair a (using *C.lunulatus* transcriptome):<br>F: 5'-AGACCGCCACCAACATCTAC<br>R: 5'-GGATGAGGGTTACGACAGGA<br><br>Pair b (using *C.lunulatus* transcriptome):<br>F: 5'-AGCACGCTACCTTTCCAGAG<br>R: 5'-CCCTTGAAGTTCTCATCCAG	R:5'- CGCAGGTTCCTGTCCTTCT	F:5'- TCATGTTCATGGCCTCCAC<br>R:5'- GCAGATCTTCAGCAGGGTGT
18S	Pair a (using *C.lunulatus* transcriptome):<br>F: 5'-GAGACTCCGGCATGCTAACT<br>R: 5'-GTAATGATCCTTCCGCAGGT	5'- ATAGTCAAGTTTGATCGTCTTCTCG	F:5'- CAGTAAGCGCGGGTCATAAG<br>R:5'- CGATCCGAGGACCTCACTAA

Supplementary Table S2: Thermal cycle parameters used to preform quantitative qPCR on *Chaetodon* butterflyfish

Gene	Enzyme mix	Cycle function	No. cycles	Temp. (°C)	Time
18S, V1aR, ITR	PerfeCTa SYBR® Green SuperMix	Initial denature	1	95	30 sec
		Denature<br>Primer anneal<br>Extension<br>+ Plate read	45	95<br>55<br>70	5 sec<br>15 sec<br>10 sec
		Melt curve<br>+ Plate read	1	65-95, 0.5 increment	5 sec
MOR, D1R	PerfeCTa SYBR® Green SuperMix	Initial denature	1	95	30 sec
		Denature<br>Primer anneal<br>Extension<br>+ Plate read	45	95<br>60<br>70	5 sec<br>15 sec<br>10 sec
		Melt curve<br>+ Plate read	1	65-95, 0.5 increment	5 sec
D2R	PerfeCTa SYBR® Green FastMix	Initial denature	1	95	3 min
		Denature<br>Primer anneal + extension<br>+ Plate read	45	95<br>60	15 sec<br>1 min
		Melt curve<br>+ Plate read	1	65-95, 0.5 increment	5 sec

Supplementary Table S3: Model summary of main and interactive effects of sex and social system on brain region-specific gene expression differences in *Chaetodon lunulatus* butterflyfishes. Results are reported as natural log-fold changes from the *a priori* comparison state of male pair bonding, with a two-tailed p value. Associated pair-wise differences between treatments (upper triangle) and their statistical significance (lower triangle) are reported for each brain region and gene, below summary statistics.

**Dm (blAMY)**	post.mean	l-95%	u-95%	eff.samp	pMCMC
geneD1R	3.142	1.8472	4.5182	1000	<0.001
geneD2R	3.6135	1.732	5.4551	1000	<0.001
geneITR	-2.7655	-5.7253	0.1035	194.148	0.016
geneMOR	2.5017	0.9068	4.0094	895.047	0.004
gener18S	14.9415	13.5903	16.2004	1000	<0.001
geneV1aR	-2.3168	-4.7494	-0.129	858.875	0.03
geneD1R:sexF	1.3124	-0.8307	3.4848	1000	0.248
geneD2R:sexF	-1.4817	-3.9562	1.4242	754.521	0.252
geneITR:sexF	-1.9483	-6.3603	2.6047	693.048	0.366
geneMOR:sexF	0.3657	-1.9418	2.7032	1000	0.758
gener18S:sexF	-0.6788	-2.5982	1.1364	1000	0.46
geneV1aR:sexF	1.1761	-1.8762	3.8531	1000	0.396
geneD1R:socialSol	-1.4655	-3.5313	0.8352	1000	0.176
geneD2R:socialSol	-1.2047	-3.6959	1.8502	1096.457	0.382
geneITR:socialSol	-193.962	-357.689	-5.2094	1.287	0.004
geneMOR:socialSol	-2.9367	-5.4574	-0.5429	892.836	0.014
gener18S:socialSol	-0.6569	-2.7192	1.4031	777.435	0.498
geneV1aR:socialSol	-1.4491	-5.0021	2.1111	1000	0.422
geneD1R:sexF:socialSol	-1.2232	-4.1923	2.2079	1000	0.428
geneD2R:sexF:socialSol	1.8539	-1.6811	6.3018	1000	0.344
geneITR:sexF:socialSol	54.4327	-139.685	217.4457	4.289	0.454
geneMOR:sexF:socialSol	1.3268	-2.0225	4.6156	1000	0.436
gener18S:sexF:socialSol	0.7555	-2.1329	3.6111	1000	0.628
geneV1aR:sexF:socialSol	-1.1577	-6.1427	4.6096	891.465	0.644

Dm (blAMY) Pair-wise p values

$D1R					
	difference				
pvalue		sexM:socialPB	sexM:socialSol	sexF:socialPB	sexF:socialSol
	sexM:socialPB	NA	-2.11433467	1.89332498	-1.985673
	sexM:socialSol	0.191114	NA	4.00765965	0.1286617
	sexF:socialPB	0.2548586	0.02636481	NA	-3.878998
	sexF:socialSol	0.1915565	0.93752835	0.02801988	NA
$D2R					
	difference				
pvalue		sexM:socialPB	sexM:socialSol	sexF:socialPB	sexF:socialSol
	sexM:socialPB	NA	-1.7380322	-2.1376927	-1.2011756
	sexM:socialSol	0.3943747	NA	-0.3996604	0.5368567
	sexF:socialPB	0.2741771	0.8453605	NA	0.9365171
	sexF:socialSol	0.5502811	0.8062066	0.6562063	NA
$ITR					
	difference				

pvalue		sexM:socialPB	sexM:socialSol	sexF:socialPB	sexF:socialSol
	sexM:socialPB	NA	-279.8274998	-2.8108276	-204.10856
	sexM:socialSol	0.1066488	NA	277.0166723	75.71894
	sexF:socialPB	0.43701	0.110682	NA	-201.29773
	sexF:socialSol	0.1361658	0.5404262	0.1417471	NA
$MOR					
	difference				
pvalue		sexM:socialPB	sexM:socialSol	sexF:socialPB	sexF:socialSol
	sexM:socialPB	NA	-4.236693617	0.5275561	-1.794922
	sexM:socialSol	0.01897804	NA	4.7642498	2.441772
	sexF:socialPB	0.75241678	0.009653445	NA	-2.322478
	sexF:socialSol	0.30748278	0.188131608	0.1757757	NA
$r18S					
	difference				
pvalue		sexM:socialPB	sexM:socialSol	sexF:socialPB	sexF:socialSol
	sexM:socialPB	NA	-0.9477639	-0.97925093	-0.8370861
	sexM:socialSol	0.5288687	NA	-0.03148701	0.1106778
	sexF:socialPB	0.4840288	0.983036	NA	0.1421648
	sexF:socialSol	0.5796442	0.9463554	0.92497113	NA
$V1aR					
	difference				
pvalue		sexM:socialPB	sexM:socialSol	sexF:socialPB	sexF:socialSol
	sexM:socialPB	NA	-2.0906618	1.6968106	-2.064007
	sexM:socialSol	0.4571102	NA	3.7874725	0.02665483
	sexF:socialPB	0.4261231	0.1733417	NA	-3.76081764
	sexF:socialSol	0.4656879	0.9937238	0.1794475	NA

**Dl (Hipp)**	post.mean	l-95%	u-95%	eff.samp	pMCMC
geneD1R	4.32E+00	2.29E+00	6.54E+00	1215.32	<0.001
geneD2R	2.16E+00	-2.01E-01	4.38E+00	752.78	0.086
geneITR	-3.25E+00	-6.07E+00	-7.73E-01	1000	0.004
geneMOR	2.98E+00	1.24E+00	4.73E+00	1000	0.002
gener18S	1.40E+01	1.23E+01	1.55E+01	1000	<0.001
geneV1aR	-2.49E+00	-4.79E+00	-4.25E-02	875.43	0.024
geneD1R:sexF	-8.71E-01	-3.31E+00	2.10E+00	1122.67	0.508
geneD2R:sexF	1.54E+00	-1.73E+00	4.64E+00	809.55	0.304
geneITR:sexF	2.46E+00	-3.04E-01	5.86E+00	1000	0.11
geneMOR:sexF	-8.96E-01	-3.29E+00	1.99E+00	897.13	0.468
gener18S:sexF	1.18E+00	-9.17E-01	3.53E+00	1000	0.298
geneV1aR:sexF	1.36E+00	-1.88E+00	4.47E+00	1000	0.358
geneD1R:socialSol	-2.20E+00	-5.29E+00	4.48E-01	1000	0.128
geneD2R:socialSol	-6.48E-03	-3.33E+00	3.16E+00	1134.11	0.958
geneITR:socialSol	-1.09E+00	-5.24E+00	3.52E+00	1000	0.634
geneMOR:socialSol	-1.36E+00	-4.07E+00	1.48E+00	1164.88	0.318
gener18S:socialSol	2.29E-01	-2.15E+00	2.66E+00	1000	0.846
geneV1aR:socialSol	-2.19E+00	-6.65E+00	2.00E+00	1000	0.286
geneD1R:sexF:socialSol	4.05E-01	-3.41E+00	4.13E+00	1000	0.8
geneD2R:sexF:socialSol	-1.06E+00	-5.49E+00	3.38E+00	1000	0.642

geneITR:sexF:socialSol	7.91E-01	-4.52E+00	5.26E+00	1000	0.734
geneMOR:sexF:socialSol	1.50E+00	-2.30E+00	5.47E+00	1000	0.45
gener18S:sexF:socialSol	-1.31E+00	-4.79E+00	1.87E+00	890.17	0.41
geneV1aR:sexF:socialSol	-6.29E+01	-1.55E+02	-4.37E-01	5.88	0.012

Dl (Hipp) Pair-wise p values

$D1R					
	difference				
pvalue		sexM:socialPB	sexM:socialSol	sexF:socialPB	sexF:socialSol
	sexM:socialPB	NA	-3.16841	-1.25707	-3.840596
	sexM:socialSol	0.134117	NA	1.911336	-0.6721889
	sexF:socialPB	0.520008	0.295636	NA	-2.5835252
	sexF:socialSol	0.057376	0.715174	0.130778	NA
$D2R					
	difference				
pvalue		sexM:socialPB	sexM:socialSol	sexF:socialPB	sexF:socialSol
	sexM:socialPB	NA	-0.00935	2.224183	0.6843199
	sexM:socialSol	0.996827	NA	2.233538	0.6936748
	sexF:socialPB	0.339648	0.352695	NA	-1.539863
	sexF:socialSol	0.765696	0.780998	0.513554	NA
$ITR					
	difference				
pvalue	sexM:socialPB	sexM:socialSol	sexF:socialPB	sexF:socialSol	
	sexM:socialPB	NA	-1.57588	3.553385	3.1190451
	sexM:socialSol	0.62438	NA	5.129269	4.6949293
	sexF:socialPB	0.124599	0.0759	NA	-0.4343394
	sexF:socialSol	0.198681	0.107248	0.828115	NA
$MOR					
	difference				
pvalue		sexM:socialPB	sexM:socialSol	sexF:socialPB	sexF:socialSol
	sexM:socialPB	NA	-1.96844	-1.2933	-1.0965406
	sexM:socialSol	0.335637	NA	0.675135	0.8718954
	sexF:socialPB	0.503268	0.746256	NA	0.1967603
	sexF:socialSol	0.576531	0.687927	0.920763	NA
$r18S					
	difference				
pvalue		sexM:socialPB	sexM:socialSol	sexF:socialPB	sexF:socialSol
	sexM:socialPB	NA	0.330311	1.701266	0.1366222
	sexM:socialSol	0.85299	NA	1.370955	-0.1936886
	sexF:socialPB	0.303461	0.435768	NA	-1.5646433
	sexF:socialSol	0.933051	0.912509	0.350976	NA
$V1aR					

	difference				
pvalue		sexM:socialPB	sexM:socialSol	sexF:socialPB	sexF:socialSol
	sexM:socialPB	NA	-3.15191	1.954299	-91.86627
	sexM:socialSol	0.318081	NA	5.106213	-88.71436
	sexF:socialPB	0.388011	0.101299	NA	-93.82057
	sexF:socialSol	0.147846	0.162791	0.138922	NA

**Vv/Vl (LS)**	post.mean	l-95%	u-95%	eff.samp	pMCMC
geneD1R	4.18263	2.05947	6.37721	1000	<0.001
geneD2R	3.43398	1.6286	5.15397	1000	<0.001
geneITR	-1.58519	-4.15107	0.43111	1000	0.158
geneMOR	2.5583	0.52161	4.40675	1000	0.012
geneV1aR	0.80748	-1.27265	2.80046	1000	0.434
gener18S	12.12274	10.80329	13.49123	1000	<0.001
geneD1R:sexF	-0.10344	-3.66066	3.3877	1000	0.98
geneD2R:sexF	0.61094	-2.46058	3.68002	1000	0.692
geneITR:sexF	-0.55273	-4.16377	3.09086	1000	0.75
geneMOR:sexF	0.1833	-3.10986	3.36018	1000	0.924
geneV1aR:sexF	-1.77035	-5.84196	1.59303	990.1	0.346
gener18S:sexF	-0.02783	-0.95233	1.11198	1000	0.962
geneD1R:socialSol	-1.80785	-4.79607	1.31936	1000	0.242
geneD2R:socialSol	0.3149	-2.46907	3.3038	1000	0.806
geneITR:socialSol	0.49331	-2.49085	3.68865	864.6	0.734
geneMOR:socialSol	0.52584	-2.27097	3.28731	1000	0.752
geneV1aR:socialSol	-3.04181	-6.09046	0.1176	1000	0.052
gener18S:socialSol	0.44774	-0.66276	1.48162	1127.8	0.406
geneD1R:sexF:socialSol	0.12635	-4.26795	4.60353	1000	0.964
geneD2R:sexF:socialSol	-1.2808	-5.1135	3.1451	1000	0.508
geneITR:sexF:socialSol	0.5128	-4.53174	5.0883	907.2	0.832
geneMOR:sexF:socialSol	-0.15703	-4.19607	4.37666	1000	0.934
geneV1aR:sexF:socialSol	3.1815	-1.61693	8.2854	961.6	0.192
gener18S:sexF:socialSol	0.33206	-0.73743	1.47798	1095.3	0.552

Vv/vl (LS) Pair-wise p values

$D1R					
	difference				
pvalue		sexM:socialPB	sexM:socialSol	sexF:socialPB	sexF:socialSol
	sexM:socialPB	NA	-2.60817	-0.14923	-2.575114
	sexM:socialSol	0.23875	NA	2.458942	0.03305623
	sexF:socialPB	0.954749	0.361308	NA	-2.4258854
	sexF:socialSol	0.232368	0.987686	0.342613	NA
$D2R					
	difference				
pvalue		sexM:socialPB	sexM:socialSol	sexF:socialPB	sexF:socialSol
	sexM:socialPB	NA	0.454304	0.881397	-0.5121025

	sexM:socialSol	0.824989	NA	0.427094	-0.9664061
	sexF:socialPB	0.701724	0.862737	NA	-1.3934996
	sexF:socialSol	0.793223	0.641384	0.535239	NA
$ITR					
	difference				
pvalue		sexM:socialPB	sexM:socialSol	sexF:socialPB	sexF:socialSol
	sexM:socialPB	NA	0.711696	-0.79742	0.65409772
	sexM:socialSol	0.763474	NA	-1.50911	-0.0575984
	sexF:socialPB	0.770038	0.584709	NA	1.45151566
	sexF:socialSol	0.778172	0.980471	0.586629	NA
$MOR					
	difference				
pvalue		sexM:socialPB	sexM:socialSol	sexF:socialPB	sexF:socialSol
	sexM:socialPB	NA	0.758632	0.264452	0.79653296
	sexM:socialSol	0.724684	NA	-0.49418	0.03790077
	sexF:socialPB	0.912191	0.834256	NA	0.53208102
	sexF:socialSol	0.695443	0.985817	0.819464	NA
$V1aR					
	difference				
pvalue		sexM:socialPB	sexM:socialSol	sexF:socialPB	sexF:socialSol
	sexM:socialPB	NA	-4.38841	-2.55408	-2.3525538
	sexM:socialSol	0.058951	NA	1.834325	2.0358532
	sexF:socialPB	0.353328	0.541294	NA	0.2015281
	sexF:socialSol	0.270102	0.426806	0.942783	NA
$r18S					
	difference				
pvalue		sexM:socialPB	sexM:socialSol	sexF:socialPB	sexF:socialSol
	sexM:socialPB	NA	0.645945	-0.04014	1.0848603
	sexM:socialSol	0.409982	NA	-0.68609	0.4389151
	sexF:socialPB	0.957733	0.540707	NA	1.1250035
	sexF:socialSol	0.371343	0.675555	0.284979	NA

**Vs (meAMY/BNST)**	post.mean	l-95%	u-95%	eff.samp	pMCMC
geneD1R	0.41519	-1.73372	2.22517	1000	0.62
geneD2R	2.66415	0.95883	4.51436	1000	0.008
geneITR	-2.7834	-5.40423	0.06744	286	0.028
geneMOR	2.0634	-2.00066	5.88315	1000	0.28
gener18S	13.8691	12.08308	15.4984	1000	<0.001
geneV1aR	-0.66775	-2.56781	1.55733	1351.4	0.514
geneD1R:sexF	4.87635	1.9087	7.92413	1000	0.006
geneD2R:sexF	0.29534	-2.23421	3.10785	1000	0.82
geneITR:sexF	1.3332	-1.86136	4.90466	766.5	0.442
geneMOR:sexF	0.48468	-4.99799	5.59678	1052	0.818

gener18S:sexF	-0.33156	-2.85742	2.0727	1000	0.796
geneV1aR:sexF	0.32751	-2.61109	3.25598	1000	0.836
geneD1R:socialSol	2.12168	-0.79227	5.04754	1000	0.16
geneD2R:socialSol	0.08216	-2.70677	3.24178	753.5	0.964
geneITR:socialSol	0.43815	-3.59942	4.77041	804.2	0.856
geneMOR:socialSol	1.33059	-4.56275	7.17892	1000	0.654
gener18S:socialSol	-0.41375	-3.33612	2.48778	855	0.762
geneV1aR:socialSol	-0.79005	-3.89873	2.71125	1000	0.644
geneD1R:sexF:socialSol	-4.64349	-8.95856	-0.44833	1000	0.032
geneD2R:sexF:socialSol	-0.54687	-4.28436	3.76456	1000	0.752
geneITR:sexF:socialSol	-1.14534	-6.60084	3.9961	680.6	0.668
geneMOR:sexF:socialSol	-0.43071	-8.13261	6.69024	1000	0.89
gener18S:sexF:socialSol	1.13119	-2.8092	5.03306	1000	0.55
geneV1aR:sexF:socialSol	-1.32989	-6.23568	3.10742	1000	0.568

Vv/vl (LS) Pair-wise p values

$D1R					
	difference				
pvalue		sexM:socialPB	sexM:socialSol	sexF:socialPB	sexF:socialSol
	sexM:socialPB	NA	3.060942	7.035079	3.3968875
	sexM:socialSol	0.162169	NA	3.974138	0.3359458
	sexF:socialPB	0.001695	0.099793	NA	-3.6381918
	sexF:socialSol	0.080558	0.876204	0.094788	NA
$D2R					
	difference				
pvalue		sexM:socialPB	sexM:socialSol	sexF:socialPB	sexF:socialSol
	sexM:socialPB	NA	0.118536	0.42608	-0.2443452
	sexM:socialSol	0.955139	NA	0.307544	-0.3628815
	sexF:socialPB	0.825598	0.885753	NA	-0.670425
	sexF:socialSol	0.897107	0.869571	0.728497	NA
$ITR					
	difference				
pvalue		sexM:socialPB	sexM:socialSol	sexF:socialPB	sexF:socialSol
	sexM:socialPB	NA	0.63212	1.923402	0.9031425
	sexM:socialSol	0.844609	NA	1.291282	0.2710224
	sexF:socialPB	0.489653	0.675387	NA	-1.0202591
	sexF:socialSol	0.75002	0.925349	0.71304	NA
$MOR					
	difference				
pvalue		sexM:socialPB	sexM:socialSol	sexF:socialPB	sexF:socialSol
	sexM:socialPB	NA	1.919634	0.699246	1.99749782
	sexM:socialSol	0.65115	NA	-1.22039	0.07786436
	sexF:socialPB	0.855757	0.765798	NA	1.29825188

	sexF:socialSol	0.608205	0.983895	0.723613	NA
$r18S					
	difference				
pvalue		sexM:socialPB	sexM:socialSol	sexF:socialPB	sexF:socialSol
	sexM:socialPB	NA	-0.59691	-0.47833	0.556718
	sexM:socialSol	0.782062	NA	0.118579	1.15363
	sexF:socialPB	0.795532	0.956062	NA	1.035051
	sexF:socialSol	0.7601	0.584902	0.580568	NA
$V1aR					
	difference				
pvalue		sexM:socialPB	sexM:socialSol	sexF:socialPB	sexF:socialSol
	sexM:socialPB	NA	-1.13979	0.472503	-2.585912
	sexM:socialSol	0.65074	NA	1.612297	-1.446117
	sexF:socialPB	0.825622	0.536849	NA	-3.058415
	sexF:socialSol	0.282817	0.591447	0.212108	NA

**Vd (NAcc)**	post.mean	l-95%	u-95%	eff.samp	pMCMC
geneD1R	3.40928	1.41427	5.25825	1000	<0.001
geneD2R	2.08561	0.45417	3.77862	1097.557	0.02
geneITR	-77.3637	-160.431	-5.46338	4.538	<0.001
geneMOR	-34.7492	-100.592	5.37739	21.038	0.078
gener18S	12.72855	11.57857	14.02614	1000	<0.001
geneV1aR	-11.2197	-40.2993	0.08154	19.051	0.01
geneD1R:sexF	-0.25354	-2.35008	2.08043	1000	0.798
geneD2R:sexF	0.51131	-2.20217	2.96635	1000	0.65
geneITR:sexF	70.11619	-3.01837	154.5589	4.295	0.018
geneMOR:sexF	11.61996	-66.1972	85.19842	59.527	0.73
gener18S:sexF	-0.17443	-1.90647	1.69883	1229.386	0.832
geneV1aR:sexF	6.64009	-8.94948	33.84802	37.513	0.262
geneD1R:socialSol	-3.63525	-6.44676	-0.7174	1303.857	0.016
geneD2R:socialSol	-2.46742	-6.27528	0.85833	1000	0.152
geneITR:socialSol	-71.8958	-256.436	91.4815	3.502	0.662
geneMOR:socialSol	15.65856	-75.4037	95.17179	70.272	0.61
gener18S:socialSol	-1.62641	-3.95243	0.54302	1000	0.144
geneV1aR:socialSol	6.18767	-10.2903	36.39662	81.08	0.424
geneD1R:sexF:socialSol	-0.49672	-3.98204	3.29604	1000	0.776
geneD2R:sexF:socialSol	-0.2799	-4.57304	4.71507	1192.098	0.87
geneITR:sexF:socialSol	-7.671	-226.221	141.0776	3.607	0.842
geneMOR:sexF:socialSol	-13.276	-126.788	105.7283	52.912	0.816
gener18S:sexF:socialSol	0.80758	-2.08886	3.62423	910.999	0.54
geneV1aR:sexF:socialSol	-11.9915	-49.4752	10.41948	32.394	0.184

Vd (NAcc) Pair-wise p values

$D1R					
	difference				
pvalue		sexM:socialPB	sexM:socialSol	sexF:socialPB	sexF:socialSol
	sexM:socialPB	NA	-5.24456	-0.36578	-6.32695
	sexM:socialSol	0.011822	NA	4.878775	-1.0824
	sexF:socialPB	0.824113	0.007921	NA	-5.96117
	sexF:socialSol	0.000638	0.597229	0.000169	NA
$D2R					
	difference				
pvalue		sexM:socialPB	sexM:socialSol	sexF:socialPB	sexF:socialSol
	sexM:socialPB	NA	-3.55973	0.737671	-3.22586
	sexM:socialSol	0.181647	NA	4.297402	0.333867
	sexF:socialPB	0.696574	0.110721	NA	-3.96354
	sexF:socialSol	0.078372	0.900303	0.041888	NA
$ITR					
	difference				
pvalue		sexM:socialPB	sexM:socialSol	sexF:socialPB	sexF:socialSol
	sexM:socialPB	NA	-103.724	101.1563	-13.6344
	sexM:socialSol	0.48642	NA	204.88	90.08936
	sexF:socialPB	0.140002	0.131735	NA	-114.791
	sexF:socialSol	0.809516	0.486853	0.065646	NA
$MOR					
	difference				
pvalue		sexM:socialPB	sexM:socialSol	sexF:socialPB	sexF:socialSol
	sexM:socialPB	NA	22.59053	16.76406	20.20134
	sexM:socialSol	0.700638	NA	-5.82647	-2.38919
	sexF:socialPB	0.7558	0.925631	NA	3.43728
	sexF:socialSol	0.645777	0.965382	0.946784	NA
$r18S					
	difference				
pvalue		sexM:socialPB	sexM:socialSol	sexF:socialPB	sexF:socialSol
	sexM:socialPB	NA	-2.34641	-0.25165	-1.43296
	sexM:socialSol	0.153677	NA	2.094758	0.913447
	sexF:socialPB	0.845353	0.216417	NA	-1.18131
	sexF:socialSol	0.251057	0.576735	0.355302	NA
$V1aR					
	difference				
pvalue		sexM:socialPB	sexM:socialSol	sexF:socialPB	sexF:socialSol
	sexM:socialPB	NA	8.926924	9.579622	1.206478
	sexM:socialSol	0.602429	NA	0.652698	-7.72045
	sexF:socialPB	0.566705	0.965746	NA	-8.37314
	sexF:socialSol	0.932346	0.591932	0.513438	NA

**POA (POA)**	post.mean	l-95%	u-95%	eff.samp	pMCMC
geneD1R	4.3673	2.6354	6.1356	1000	<0.001
geneD2R	3.8541	2.2052	5.5648	1000	<0.001
geneITR	-0.5018	-2.1461	1.2122	904.4	0.594
geneMOR	1.9263	-0.1851	3.7134	1000	0.056
geneV1aR	-1.2953	-3.3395	0.659	1031.2	0.212
gener18S	13.06	11.929	14.1965	1000	<0.001
geneD1R:sexF	0.8944	-1.4561	3.1279	1000	0.466
geneD2R:sexF	1.2108	-0.9323	3.3259	1000	0.29
geneITR:sexF	0.8153	-1.3922	3.1205	1000	0.52
geneMOR:sexF	2.4464	-0.4033	4.8277	1000	0.068
geneV1aR:sexF	2.7253	0.1442	5.4334	1000	0.04
gener18S:sexF	0.3687	-0.5607	1.4214	1000	0.46
geneD1R:socialSol	-3.2855	-6.2908	-0.6255	1000	0.028
geneD2R:socialSol	-1.339	-3.7868	1.2976	1096	0.3
geneITR:socialSol	-1.7395	-4.4291	1.3362	1000	0.222
geneMOR:socialSol	0.4157	-2.3244	3.0698	1000	0.75
geneV1aR:socialSol	0.1232	-2.463	3.7451	1000	0.938
gener18S:socialSol	-0.3185	-1.3934	0.5331	1000	0.53
geneD1R:sexF:socialSol	1.6993	-2.0011	5.0748	1195.5	0.356
geneD2R:sexF:socialSol	0.468	-3.2633	3.681	1258.1	0.782
geneITR:sexF:socialSol	1.8269	-1.978	5.4142	1000	0.344
geneMOR:sexF:socialSol	-0.5078	-3.8877	3.2985	1000	0.786
geneV1aR:sexF:socialSol	-0.9352	-4.7723	2.5485	1000	0.662
gener18S:sexF:socialSol	0.2283	-0.7923	1.3007	1000	0.66

POA (POA) Pair-wise p values

$D1R					
	difference				
pvalue		sexM:socialPB	sexM:socialSol	sexF:socialPB	sexF:socialSol
	sexM:socialPB	NA	-4.74004	1.290324	-0.99813
	sexM:socialSol	0.024582	NA	6.0303639	3.741906
	sexF:socialPB	0.448421	0.002152	NA	-2.28846
	sexF:socialSol	0.581531	0.057079	0.1702788	NA
$D2R					
	difference				
pvalue		sexM:socialPB	sexM:socialSol	sexF:socialPB	sexF:socialSol
	sexM:socialPB	NA	-1.93176	1.7468499	0.490215
	sexM:socialSol	0.311711	NA	3.6786112	2.421976
	sexF:socialPB	0.278293	0.031272	NA	-1.25664
	sexF:socialSol	0.775446	0.190447	0.4355411	NA
$ITR					
	difference				

pvalue		sexM:socialPB	sexM:socialSol	sexF:socialPB	sexF:socialSol
	sexM:socialPB	NA	-2.50952	1.1762082	1.302365
	sexM:socialSol	0.224518	NA	3.6857259	3.811883
	sexF:socialPB	0.498062	0.073931	NA	0.126157
	sexF:socialSol	0.472984	0.079209	0.9425484	NA
$MOR					
	difference				
pvalue		sexM:socialPB	sexM:socialSol	sexF:socialPB	sexF:socialSol
	sexM:socialPB	NA	0.599729	3.5294268	3.396511
	sexM:socialSol	0.765904	NA	2.9296983	2.796782
	sexF:socialPB	0.059922	0.100699	NA	-0.13292
	sexF:socialSol	0.067231	0.136976	0.9366706	NA
$V1aR					
	difference				
pvalue		sexM:socialPB	sexM:socialSol	sexF:socialPB	sexF:socialSol
	sexM:socialPB	NA	0.177783	3.9317056	2.760335
	sexM:socialSol	0.936343	NA	3.7539222	2.582551
	sexF:socialPB	0.04414	0.073261	NA	-1.17137
	sexF:socialSol	0.180124	0.217466	0.5176704	NA
$r18S					
	difference				
pvalue		sexM:socialPB	sexM:socialSol	sexF:socialPB	sexF:socialSol
	sexM:socialPB	NA	-0.45956	0.5318711	0.401606
	sexM:socialSol	0.528481	NA	0.9914331	0.861168
	sexF:socialPB	0.463492	0.329933	NA	-0.13027
	sexF:socialSol	0.727028	0.383092	0.8933919	NA

**Vc (Str/CP)**	post.mean	l-95%	u-95%	eff.samp	pMCMC
geneD1R	3.83523	1.73107	6.17979	1000	0.004
geneD2R	3.08301	1.39313	4.83215	1024.111	0.004
geneITR	-0.66475	-2.66405	1.2628	1000	0.502
geneMOR	0.02413	-4.56867	3.78887	707.354	0.924
gener18S	12.72933	10.73039	14.39361	901.265	<0.001
geneV1aR	-2.39528	-7.21094	1.97533	487.252	0.192
geneD1R:sexF	-0.28664	-2.97676	2.8047	1000	0.83
geneD2R:sexF	1.32038	-1.04093	3.61791	1073.205	0.294
geneITR:sexF	-0.66109	-3.3408	2.2858	1000	0.694
geneMOR:sexF	0.8442	-5.30885	7.01668	617.27	0.728
gener18S:sexF	0.34107	-2.50415	2.66242	1000	0.814
geneV1aR:sexF	-374.95525	-727.11558	-2.64809	1.614	<0.001
geneD1R:socialSol	-1.32173	-4.372	1.27787	1223.358	0.324
geneD2R:socialSol	0.73425	-1.64315	2.94601	929.199	0.554
geneITR:socialSol	0.26318	-2.42858	3.1579	893.813	0.872
geneMOR:socialSol	1.66246	-4.39772	7.06733	1000	0.46

gener18S:socialSol	0.14103	-2.14284	2.68265	867.14	0.912
geneV1aR:socialSol	-0.41867	-6.27297	5.60197	763.458	0.862
geneD1R:sexF:socialSol	0.9841	-2.6053	4.49957	1000	0.566
geneD2R:sexF:socialSol	-1.66887	-4.9295	1.42726	1000	0.282
geneITR:sexF:socialSol	1.29786	-2.33542	5.18657	878.854	0.49
geneMOR:sexF:socialSol	1.19054	-7.03369	9.27663	638.672	0.766
gener18S:sexF:socialSol	0.43835	-3.4251	3.29374	1000	0.802
geneV1aR:sexF:socialSol	375.32016	4.42487	728.01143	1.721	<0.001

Vc (Str.CP) Pair-wise p values

$D1R					
	difference				
pvalue		sexM:socialPB	sexM:socialSol	sexF:socialPB	sexF:socialSol
	sexM:socialPB	NA	-1.90685	-0.41353	-0.90062
	sexM:socialSol	0.351856	NA	1.4933177	1.00623
	sexF:socialPB	0.840991	0.430851	NA	-0.48709
	sexF:socialSol	0.639282	0.54399	0.7721882	NA
$D2R					
	difference				
pvalue		sexM:socialPB	sexM:socialSol	sexF:socialPB	sexF:socialSol
	sexM:socialPB	NA	1.059301	1.9049018	0.556533
	sexM:socialSol	0.543706	NA	0.8456005	-0.50277
	sexF:socialPB	0.279922	0.641229	NA	-1.34837
	sexF:socialSol	0.733316	0.755973	0.3928179	NA
$ITR					
	difference				
pvalue		sexM:socialPB	sexM:socialSol	sexF:socialPB	sexF:socialSol
	sexM:socialPB	NA	0.37969	-0.9537568	1.298349
	sexM:socialSol	0.858353	NA	-1.3334468	0.918659
	sexF:socialPB	0.647843	0.535344	NA	2.252106
	sexF:socialSol	0.48605	0.617881	0.232533	NA
$MOR					
	difference				
pvalue		sexM:socialPB	sexM:socialSol	sexF:socialPB	sexF:socialSol
	sexM:socialPB	NA	2.39842	1.2179173	5.333917
	sexM:socialSol	0.553849	NA	-1.1805031	2.935497
	sexF:socialPB	0.780032	0.779456	NA	4.116
	sexF:socialSol	0.155075	0.439936	0.3113202	NA
$r18S					
	difference				
pvalue		sexM:socialPB	sexM:socialSol	sexF:socialPB	sexF:socialSol
	sexM:socialPB	NA	0.203459	0.4920597	1.32792

	sexM:socialSol	0.910904	NA	0.2886004	1.124461
	sexF:socialPB	0.793376	0.878347	NA	0.83586
	sexF:socialSol	0.431022	0.500693	0.6291014	NA
$V1aR					
	difference				
pvalue		sexM:socialPB	sexM:socialSol	sexF:socialPB	sexF:socialSol
	sexM:socialPB	NA	-0.60402	-540.94607	-0.07755
	sexM:socialSol	0.915685	NA	-540.34206	0.526464
	sexF:socialPB	0.10121	0.101881	NA	540.8685
	sexF:socialSol	0.989267	0.924626	0.1012361	NA

**TPp (VTA)**	post.mean	l-95%	u-95%	eff.samp	pMCMC
geneD1R	2.04395	0.19165	4.39896	1000	0.054
geneD2R	2.74824	0.98103	4.55404	600.48	0.006
geneITR	-1.82547	-3.92205	0.5342	1000	0.092
geneMOR	1.52105	-0.27599	3.50026	1000	0.114
gener18S	12.27213	10.38986	13.76381	1000	<0.001
geneV1aR	-6.93717	-19.51797	0.31516	66.53	0.016
geneD1R:sexF	1.83103	-1.22826	4.87981	1000	0.252
geneD2R:sexF	-0.01939	-2.60002	2.54214	1000	0.988
geneITR:sexF	0.50735	-2.68307	3.5884	1000	0.75
geneMOR:sexF	-0.16825	-2.85415	2.48163	1328.47	0.922
gener18S:sexF	-0.496	-2.62555	2.01648	1051.41	0.714
geneV1aR:sexF	3.63786	-6.45656	14.81306	350.27	0.306
geneD1R:socialSol	-0.47507	-3.76198	2.72547	992.33	0.772
geneD2R:socialSol	0.26083	-2.5545	3.64153	884.18	0.886
geneITR:socialSol	0.79589	-2.78697	3.75057	1000	0.638
geneMOR:socialSol	1.03883	-2.15066	4.12994	1202.87	0.544
gener18S:socialSol	0.90583	-1.96286	3.70162	1000	0.514
geneV1aR:socialSol	3.16256	-7.2433	15.28961	263.38	0.428
geneD1R:sexF:socialSol	-2.76729	-6.94053	1.54022	1000	0.18
geneD2R:sexF:socialSol	-0.42801	-4.29555	3.77439	973.19	0.832
geneITR:sexF:socialSol	-0.56864	-5.02669	3.60872	1000	0.794
geneMOR:sexF:socialSol	-1.15963	-5.55014	3.05552	1000	0.602
gener18S:sexF:socialSol	0.15373	-3.27617	4.00776	1000	0.95
geneV1aR:sexF:socialSol	-1.28967	-14.42093	15.54396	1009.13	0.798

TPp (VTA) Pair-wise p values

$D1R					
	difference				
pvalue		sexM:socialPB	sexM:socialSol	sexF:socialPB	sexF:socialSol
	sexM:socialPB	NA	-0.68537	2.64162402	-2.03611
	sexM:socialSol	0.764073	NA	3.32699799	-1.35073
	sexF:socialPB	0.249946	0.174	NA	-4.67773

	sexF:socialSol	0.341659	0.543793	0.03582548	NA
$D2R					
	difference				
pvalue		sexM:socialPB	sexM:socialSol	sexF:socialPB	sexF:socialSol
	sexM:socialPB	NA	0.376301	-0.0279669	-0.26916
	sexM:socialSol	0.867176	NA	-0.4042675	-0.64546
	sexF:socialPB	0.988275	0.860694	NA	-0.24119
	sexF:socialSol	0.886955	0.776691	0.897646	NA
$ITR					
	difference				
pvalue		sexM:socialPB	sexM:socialSol	sexF:socialPB	sexF:socialSol
	sexM:socialPB	NA	1.148229	0.7319514	1.059805
	sexM:socialSol	0.630556	NA	-0.4162777	-0.08842
	sexF:socialPB	0.752724	0.85878	NA	0.327854
	sexF:socialSol	0.628487	0.968866	0.8778985	NA
$MOR					
	difference				
pvalue		sexM:socialPB	sexM:socialSol	sexF:socialPB	sexF:socialSol
	sexM:socialPB	NA	1.49871	-0.2427319	-0.41702
	sexM:socialSol	0.52137	NA	-1.741442	-1.91573
	sexF:socialPB	0.904496	0.454887	NA	-0.17429
	sexF:socialSol	0.83267	0.412547	0.9343204	NA
$r18S					
	difference				
pvalue		sexM:socialPB	sexM:socialSol	sexF:socialPB	sexF:socialSol
	sexM:socialPB	NA	1.306836	-0.7155704	0.813057
	sexM:socialSol	0.527847	NA	-2.0224059	-0.49378
	sexF:socialPB	0.687863	0.345876	NA	1.528628
	sexF:socialSol	0.654031	0.814344	0.3975562	NA
$V1aR					
	difference				
pvalue		sexM:socialPB	sexM:socialSol	sexF:socialPB	sexF:socialSol
	sexM:socialPB	NA	4.562612	5.248318	7.950331
	sexM:socialSol	0.591138	NA	0.6857063	3.38772
	sexF:socialPB	0.518361	0.924113	NA	2.702013
	sexF:socialSol	0.343416	0.640093	0.6837805	NA

Key: post. mean = posterior mean, l-95% = lower 95% credible interval limit, u-95%= upper 95% credible interval limit, eff.samp = effective sample size, pMCMC = Bayesian two-tailed p-value at alpha = 0.05, *Brain region abbreviations:* teleost: telen. = telencephalon, Dm = medial part of the dorsal telen., Vd = dorsal part of the ventral telen., Dl = lateral part of the dorsal telen., Vv/Vl = lateral and ventral part of the ventral telen., Vs = supracommissural part of the ventral telen., Vc = central part of the ventral telen., POA = pre optic area, TPp = periventricular part of the posterior tuberculum., putative mammalian homolog: blAMY = basolateral amygdala, NAcc = nucleus accumbens, HIP = hippocampus, LS = lateral septum, meAMY/BNST = medial amygdala/bed nucleus of the stria terminalis, Str = Striatum, CP = caudate putamen, VTA = ventral tegmental area.

Supplementary Table S4: Model summary of main and interactive effects of sex and social system on brain region-specific gene expression differences in *Chaetodon* butterflyfish species. Results are reported as natural log-fold changes from the *a priori* comparison state of male pair bonding, with a two-tailed p value. Associated pair-wise differences between treatments (upper triangle) and their statistical significance (lower triangle) are reported for each brain region and gene, below summary statistics.

**Dm (blAMY)**	post.mean	l-95%	u-95%	eff.samp	pMCMC
geneD1R	1.44574	0.22494	2.68997	1000	0.038
geneD2R	2.62249	1.81163	3.44395	1000	<0.001
geneITR	-3.10927	-4.28152	-1.80564	1000	<0.001
geneMOR	1.70223	0.58824	2.83608	1000	0.006
gener18S	13.53278	12.80904	14.14786	1000	<0.001
geneV1aR	-1.05394	-1.96855	-0.09502	1000	0.022
geneD1R:sexF	-0.14586	-1.88106	1.66529	1000	0.87
geneD2R:sexF	-0.28945	-1.42643	0.96102	817.5	0.618
geneITR:sexF	-0.02948	-1.84844	1.62388	1000	0.944
geneMOR:sexF	0.0985	-1.55249	1.61293	1000	0.91
gener18S:sexF	0.0779	-0.76861	0.84981	1000	0.852
geneV1aR:sexF	1.00629	-0.13455	2.38914	1000	0.108
geneD1R:socialSol	2.23853	-0.914	5.52428	1000	0.13
geneD2R:socialSol	-0.57246	-2.71433	1.72687	1000	0.622
geneITR:socialSol	2.13715	-0.41862	4.80307	941.7	0.1
geneMOR:socialSol	1.36213	-1.71427	4.11056	1000	0.35
gener18S:socialSol	0.47527	-0.38195	1.28566	1242.6	0.27
geneV1aR:socialSol	-2.91336	-6.60431	0.63693	1000	0.088
geneD1R:sexF:socialSol	-0.44821	-4.06032	2.83105	790.7	0.8
geneD2R:sexF:socialSol	0.73804	-1.9297	2.91302	1000	0.56
geneITR:sexF:socialSol	-2.90295	-6.06224	0.42407	808.7	0.064
geneMOR:sexF:socialSol	-1.03257	-4.24476	2.07272	909.7	0.522
gener18S:sexF:socialSol	0.36122	-0.42896	1.26745	1000	0.426
geneV1aR:sexF:socialSol	0.79212	-3.12559	4.80783	1000	0.718

Dm (blAMY) Pair-wise p values

$D1R					
	difference				
pvalue		sexM:socialPB	sexM:socialSol	sexF:socialPB	sexF:socialSol
	sexM:socialPB	NA	3.22952	-0.21043	2.372465
	sexM:socialSol	0.154205	NA	-3.43995	-0.85706
	sexF:socialPB	0.873949	0.153178	NA	2.58289
	sexF:socialSol	0.036305	0.700799	0.02824	NA
$D2R					
	difference				

pvalue		sexM:socialPB	sexM:socialSol	sexF:socialPB	sexF:socialSol
	sexM:socialPB	NA	-0.82588	-0.41759	-0.17871
	sexM:socialSol	0.613352	NA	0.408292	0.647172
	sexF:socialPB	0.632868	0.806075	NA	0.23888
	sexF:socialSol	0.822765	0.686071	0.761563	NA
$ITR					
	difference				
pvalue		sexM:socialPB	sexM:socialSol	sexF:socialPB	sexF:socialSol
	sexM:socialPB	NA	3.083252	-0.04253	-1.14735
	sexM:socialSol	0.107639	NA	-3.12578	-4.23061
	sexF:socialPB	0.973035	0.102134	NA	-1.10482
	sexF:socialSol	0.354609	0.029861	0.365888	NA
$MOR					
	difference				
pvalue		sexM:socialPB	sexM:socialSol	sexF:socialPB	sexF:socialSol
	sexM:socialPB	NA	1.965144	0.142103	0.617563
	sexM:socialSol	0.359579	NA	-1.82304	-1.34758
	sexF:socialPB	0.902547	0.391523	NA	0.475459
	sexF:socialSol	0.556523	0.506906	0.64999	NA
$r18S					
	difference				
pvalue		sexM:socialPB	sexM:socialSol	sexF:socialPB	sexF:socialSol
	sexM:socialPB	NA	0.685669	0.112393	1.319196
	sexM:socialSol	0.266585	NA	-0.57328	0.633528
	sexF:socialPB	0.846544	0.522219	NA	1.206804
	sexF:socialSol	0.044211	0.396899	0.046865	NA
$V1aR					
	difference				
pvalue		sexM:socialPB	sexM:socialSol	sexF:socialPB	sexF:socialSol
	sexM:socialPB	NA	-4.20309	1.451776	-1.60853
	sexM:socialSol	0.120428	NA	5.654864	2.594563
	sexF:socialPB	0.12302	0.032155	NA	-3.0603
	sexF:socialSol	0.079061	0.331076	0.000561	NA

**DI (Hip)**	post.mean	l-95%	u-95%	eff.samp	pMCMC
geneD1R	2.83603	2.00926	3.65276	1000	<0.001
geneD2R	3.88142	3.03286	4.7382	1000	<0.001
geneITR	-1.32316	-2.23486	-0.3028	726.7	0.012
geneMOR	2.77672	1.94175	3.55915	1000	<0.001
gener18S	13.60635	12.95256	14.25684	1000	<0.001
geneV1aR	-1.62401	-2.58845	-0.5703	1000	<0.001
geneD1R:sexF	-0.54235	-1.76821	0.54665	1000	0.378
geneD2R:sexF	-0.31623	-1.41107	0.93808	1000	0.574
geneITR:sexF	0.48435	-0.91787	1.65968	1000	0.448
geneMOR:sexF	-0.45118	-1.49802	0.68995	1000	0.416
gener18S:sexF	0.02251	-0.67669	0.8399	1000	0.976
geneV1aR:sexF	0.1891	-1.31632	1.48655	1000	0.794
geneD1R:socialSol	-2.22454	-4.0422	-0.19688	1000	0.014
geneD2R:socialSol	-1.12374	-3.29766	0.73992	1000	0.284
geneITR:socialSol	-0.8278	-3.11753	1.62734	1000	0.486
geneMOR:socialSol	-0.40694	-2.23843	1.65738	717.5	0.672
gener18S:socialSol	0.61907	-0.21653	1.396	1000	0.152
geneV1aR:socialSol	-1.08298	-3.68386	1.43255	1000	0.43
geneD1R:sexF:socialSol	3.00343	0.84556	5.27253	1118.6	0.01
geneD2R:sexF:socialSol	1.21415	-1.08482	3.64262	1000	0.314
geneITR:sexF:socialSol	0.97589	-1.76516	3.73528	1000	0.474
geneMOR:sexF:socialSol	0.89322	-1.29719	2.98213	891.2	0.424
gener18S:sexF:socialSol	0.65615	-0.29263	1.47405	867.5	0.166
geneV1aR:sexF:socialSol	1.50322	-1.33647	4.45025	1000	0.316

DI (Hip) Pair-wise p values

$D1R					
	difference				
pvalue		sexM:socialPB	sexM:socialSol	sexF:socialPB	sexF:socialSol
	sexM:socialPB	NA	-3.20933	-0.78244	0.341253
	sexM:socialSol	0.026028	NA	2.426885	3.550583
	sexF:socialPB	0.37126	0.102441	NA	1.123698
	sexF:socialSol	0.670096	0.012692	0.158273	NA
$D2R					
	difference				
pvalue		sexM:socialPB	sexM:socialSol	sexF:socialPB	sexF:socialSol
	sexM:socialPB	NA	-1.62122	-0.45622	-0.32579
	sexM:socialSol	0.289302	NA	1.164997	1.29543
	sexF:socialPB	0.60902	0.444812	NA	0.130432
	sexF:socialSol	0.694837	0.396618	0.87223	NA
$ITR					
	difference				

pvalue		sexM:socialPB	sexM:socialSol	sexF:socialPB	sexF:socialSol
	sexM:socialPB	NA	-1.19426	0.698769	0.912419
	sexM:socialSol	0.508303	NA	1.89303	2.10668
	sexF:socialPB	0.461424	0.298583	NA	0.21365
	sexF:socialSol	0.309439	0.242602	0.806035	NA
$MOR					
	difference				
pvalue		sexM:socialPB	sexM:socialSol	sexF:socialPB	sexF:socialSol
	sexM:socialPB	NA	-0.58709	-0.65091	0.050645
	sexM:socialSol	0.685328	NA	-0.06382	0.637739
	sexF:socialPB	0.412403	0.964847	NA	0.701555
	sexF:socialSol	0.948753	0.654671	0.360568	NA
$r18S					
	difference				
pvalue		sexM:socialPB	sexM:socialSol	sexF:socialPB	sexF:socialSol
	sexM:socialPB	NA	0.893134	0.032469	1.872228
	sexM:socialSol	0.139886	NA	-0.86067	0.979094
	sexF:socialPB	0.953443	0.290072	NA	1.83976
	sexF:socialSol	0.005993	0.17752	0.002714	NA
$V1aR					
	difference				
pvalue		sexM:socialPB	sexM:socialSol	sexF:socialPB	sexF:socialSol
	sexM:socialPB	NA	-1.56241	0.272819	0.879098
	sexM:socialSol	0.422236	NA	1.835228	2.441507
	sexF:socialPB	0.79077	0.347525	NA	0.606279
	sexF:socialSol	0.328776	0.201258	0.514288	NA

**Vv/vl (LS)**	post.mean	l-95%	u-95%	eff.samp	pMCMC
geneD1R	3.5832	2.489433	4.775977	1000	<0.001
geneD2R	5.298092	4.186972	6.382995	1000	<0.001
geneITR	0.609692	-0.59512	1.670394	1764.69	0.314
geneMOR	4.973526	3.787024	6.112364	1000	<0.001
gener18S	13.55028	12.44062	14.57589	713.78	<0.001
geneV1aR	-0.51312	-1.78853	0.615867	1000	0.41
geneD1R:sexF	-0.73147	-2.48378	0.909196	907.46	0.382
geneD2R:sexF	-0.74199	-2.4489	0.894267	1608.73	0.37
geneITR:sexF	-1.79562	-3.86023	-0.05581	1000	0.06
geneMOR:sexF	-1.60171	-3.40637	0.194791	843.32	0.074
gener18S:sexF	-1.11077	-2.77129	0.590953	893.51	0.184
geneV1aR:sexF	-0.00968	-2.03937	1.677377	864.35	0.99
geneD1R:socialSol	-0.98122	-4.56126	2.151644	1000	0.568
geneD2R:socialSol	-1.60931	-4.97809	0.845963	1000	0.248
geneITR:socialSol	-21.8665	-50.8821	-2.58244	18.94	0.006
geneMOR:socialSol	-2.22592	-4.99823	0.953448	988.09	0.144

gener18S:socialSol	0.405981	-2.47623	3.58375	1000	0.8
geneV1aR:socialSol	-0.02057	-3.00152	3.337803	780.22	0.962
geneD1R:sexF:socialSol	0.941765	-2.72746	4.455502	911.94	0.61
geneD2R:sexF:socialSol	0.110958	-2.98804	3.564392	1000	0.944
geneITR:sexF:socialSol	21.1941	1.530546	50.45012	18.98	0.01
geneMOR:sexF:socialSol	1.219434	-2.39012	4.438878	1000	0.488
gener18S:sexF:socialSol	0.617217	-2.84638	4.008031	1000	0.72
geneV1aR:sexF:socialSol	-0.71249	-4.06526	3.532017	822.51	0.734

Vv/vl (LS) Pair-wise p values

$D1R					
	difference				
pvalue		sexM:socialPB	sexM:socialSol	sexF:socialPB	sexF:socialSol
	sexM:socialPB	NA	-1.11199	-1.00306	-1.12236
	sexM:socialSol	0.652415	NA	0.108926	-0.01038
	sexF:socialPB	0.433234	0.965808	NA	-0.1193
	sexF:socialSol	0.310196	0.99668	0.915758	NA
$D2R					
	difference				
pvalue		sexM:socialPB	sexM:socialSol	sexF:socialPB	sexF:socialSol
	sexM:socialPB	NA	-2.38907	-1.10505	-3.26143
	sexM:socialSol	0.275636	NA	1.284018	-0.87236
	sexF:socialPB	0.380425	0.581213	NA	-2.15637
	sexF:socialSol	0.002987	0.694601	0.060225	NA
$ITR					
	difference				
pvalue		sexM:socialPB	sexM:socialSol	sexF:socialPB	sexF:socialSol
	sexM:socialPB	NA	-69.5825	-2.69084	-3.49217
	sexM:socialSol	0.03968	NA	66.89166	66.09033
	sexF:socialPB	0.057969	0.048714	NA	-0.80133
	sexF:socialSol	0.002544	0.050417	0.566333	NA
$MOR					
	difference				
pvalue		sexM:socialPB	sexM:socialSol	sexF:socialPB	sexF:socialSol
	sexM:socialPB	NA	-3.25798	-2.38402	-3.78774
	sexM:socialSol	0.125114	NA	0.87396	-0.52976
	sexF:socialPB	0.071924	0.69984	NA	-1.40372
	sexF:socialSol	0.001037	0.805827	0.288721	NA
$r18S					
	difference				
pvalue		sexM:socialPB	sexM:socialSol	sexF:socialPB	sexF:socialSol
	sexM:socialPB	NA	0.361916	-1.74085	-0.18854
	sexM:socialSol	0.861812	NA	-2.10276	-0.55045

	sexF:socialPB	0.17367	0.340323	NA	1.552312
	sexF:socialSol	0.863452	0.793924	0.212397	NA
$V1aR					
	difference				
pvalue		sexM:socialPB	sexM:socialSol	sexF:socialPB	sexF:socialSol
	sexM:socialPB	NA	-0.01282	0.086331	-0.97882
	sexM:socialSol	0.995491	NA	0.099154	-0.966
	sexF:socialPB	0.950805	0.9667	NA	-1.06516
	sexF:socialSol	0.41617	0.673975	0.416138	NA

**Vs (meAMY/BNST)**	post.mean	l-95%	u-95%	eff.samp	pMCMC
geneD1R	-0.02012	-1.58107	1.27648	1000	0.986
geneD2R	2.7909	1.7242	3.72432	1000	<0.001
geneITR	-1.78011	-2.90743	-0.63882	1000	<0.001
geneMOR	3.82531	2.21181	5.19858	1000	<0.001
gener18S	12.93983	12.22084	13.65862	1000	<0.001
geneV1aR	-0.20625	-1.17062	0.81149	1000	0.706
geneD1R:sexF	0.48622	-1.2947	2.55869	1280.198	0.622
geneD2R:sexF	-1.07103	-2.5067	0.57119	1000	0.17
geneITR:sexF	-0.34163	-2.0711	1.24912	1000	0.688
geneMOR:sexF	-1.39005	-3.42641	0.63305	890.407	0.174
gener18S:sexF	-0.14552	-1.00938	0.6635	1000	0.74
geneV1aR:sexF	-1.20378	-2.65877	0.29088	1134.831	0.116
geneD1R:socialSol	2.28408	-0.82678	4.89858	1000	0.122
geneD2R:socialSol	-0.80521	-3.36659	1.63126	1000	0.546
geneITR:socialSol	-62.7912	-107.403	-10.6826	6.166	<0.001
geneMOR:socialSol	-2.142	-5.12538	1.26886	825.33	0.19
gener18S:socialSol	0.25603	-0.57557	1.12704	1044.283	0.58
geneV1aR:socialSol	-2.48793	-5.46044	0.27616	1110.833	0.094
geneD1R:sexF:socialSol	-1.69031	-4.90092	1.47637	1141.286	0.324
geneD2R:sexF:socialSol	0.50767	-2.18516	3.55699	1000	0.732
geneITR:sexF:socialSol	61.62759	9.03708	106.7488	6.172	0.002
geneMOR:sexF:socialSol	1.68438	-2.25177	5.23685	951.973	0.352
gener18S:sexF:socialSol	0.4844	-0.47065	1.47312	1000	0.266
geneV1aR:sexF:socialSol	0.39478	-2.4252	4.16885	1145.064	0.826

Vs (meAMY/BNST) Pair-wise p values

$D1R					
	difference				
pvalue		sexM:socialPB	sexM:socialSol	sexF:socialPB	sexF:socialSol
	sexM:socialPB	NA	3.295237	0.70147	1.5581
	sexM:socialSol	0.121654	NA	-2.59377	-1.73714
	sexF:socialPB	0.630149	0.254291	NA	0.85663
	sexF:socialSol	0.216014	0.393439	0.514274	NA

$D2R					
	difference				
pvalue		sexM:socialPB	sexM:socialSol	sexF:socialPB	sexF:socialSol
	sexM:socialPB	NA	-1.16167	-1.54517	-1.97443
	sexM:socialSol	0.537387	NA	-0.3835	-0.81276
	sexF:socialPB	0.170544	0.842261	NA	-0.42926
	sexF:socialSol	0.040983	0.659846	0.669985	NA
$ITR					
	difference				
pvalue		sexM:socialPB	sexM:socialSol	sexF:socialPB	sexF:socialSol
	sexM:socialPB	NA	-90.5886	-0.49287	-2.17165
	sexM:socialSol	0.017358	NA	90.09572	88.41694
	sexF:socialPB	0.689377	0.017821	NA	-1.67878
	sexF:socialSol	0.064221	0.020139	0.157218	NA
$MOR					
	difference				
pvalue		sexM:socialPB	sexM:socialSol	sexF:socialPB	sexF:socialSol
	sexM:socialPB	NA	-3.09025	-2.00542	-2.66562
	sexM:socialSol	0.199156	NA	1.084839	0.424631
	sexF:socialPB	0.189659	0.655901	NA	-0.66021
	sexF:socialSol	0.052704	0.852147	0.625882	NA
$r18S					
	difference				
pvalue		sexM:socialPB	sexM:socialSol	sexF:socialPB	sexF:socialSol
	sexM:socialPB	NA	0.369375	-0.20994	0.858281
	sexM:socialSol	0.57556	NA	-0.57931	0.488907
	sexF:socialPB	0.734772	0.535833	NA	1.068219
	sexF:socialSol	0.231286	0.515069	0.118097	NA
$V1aR					
	difference				
pvalue		sexM:socialPB	sexM:socialSol	sexF:socialPB	sexF:socialSol
	sexM:socialPB	NA	-3.58932	-1.73669	-4.75646
	sexM:socialSol	0.095338	NA	1.852629	-1.16714
	sexF:socialPB	0.111333	0.389553	NA	-3.01977
	sexF:socialSol	3.8E-05	0.592622	0.008905	NA

**Vd (NAcc)**	post.mean	l-95%	u-95%	eff.samp	pMCMC
geneD1R	0.030583	-1.05863	1.195641	858.5	0.934
geneD2R	1.045259	0.103456	2.026887	1000	0.044
geneITR	-6.38492	-9.32547	-3.45683	453.3	<0.001
geneMOR	-0.37873	-2.15279	1.173196	1000	0.7
gener18S	11.49026	10.80712	12.08588	1000	<0.001
geneV1aR	-3.44397	-4.94704	-2.00824	1000	<0.001

geneD1R:sexF	1.165669	-0.3764	2.755153	1000	0.154
geneD2R:sexF	-0.16332	-1.83136	1.149953	1000	0.846
geneITR:sexF	2.356562	-0.48914	6.131001	599.6	0.118
geneMOR:sexF	-0.68067	-3.41874	1.952773	888.1	0.622
gener18S:sexF	-0.00686	-0.70288	0.791048	1000	0.986
geneV1aR:sexF	0.323769	-1.66843	2.121991	1000	0.738
geneD1R:socialSol	0.474709	-1.65255	2.985605	1000	0.706
geneD2R:socialSol	0.22745	-2.31579	2.317808	1000	0.812
geneITR:socialSol	2.169236	-2.24577	6.639113	539.8	0.338
geneMOR:socialSol	0.74441	-2.70441	4.278152	1000	0.726
gener18S:socialSol	0.332802	-0.47689	1.248549	1000	0.444
geneV1aR:socialSol	1.105303	-1.67422	3.719492	1036.7	0.444
geneD1R:sexF:socialSol	-2.02674	-4.69551	0.557911	1000	0.128
geneD2R:sexF:socialSol	-1.38164	-3.90727	1.651573	1000	0.322
geneITR:sexF:socialSol	-2.46767	-7.37543	2.770347	703.5	0.336
geneMOR:sexF:socialSol	0.117548	-4.53717	4.430892	1000	0.932
gener18S:sexF:socialSol	0.349958	-0.59411	1.223655	1000	0.454
geneV1aR:sexF:socialSol	-2.04997	-5.42774	1.009508	995.5	0.21

Vd (NAcc) Pair-wise p values

$D1R					
	difference				
pvalue		sexM:socialPB	sexM:socialSol	sexF:socialPB	sexF:socialSol
	sexM:socialPB	NA	0.805892	1.706549	-0.61999
	sexM:socialSol	0.639216	NA	0.900657	-1.42588
	sexF:socialPB	0.137	0.606262	NA	-2.32654
	sexF:socialSol	0.547509	0.388731	0.03047	NA
$D2R					
	difference				
pvalue		sexM:socialPB	sexM:socialSol	sexF:socialPB	sexF:socialSol
	sexM:socialPB	NA	0.259607	-0.29081	-1.90389
	sexM:socialSol	0.875115	NA	-0.55042	-2.16349
	sexF:socialPB	0.798517	0.754715	NA	-1.61307
	sexF:socialSol	0.054982	0.188008	0.141152	NA
$ITR					
	difference				
pvalue		sexM:socialPB	sexM:socialSol	sexF:socialPB	sexF:socialSol
	sexM:socialPB	NA	3.129402	3.432013	3.089294
	sexM:socialSol	0.348683	NA	0.302611	-0.04011
	sexF:socialPB	0.165197	0.914274	NA	-0.34272
	sexF:socialSol	0.19753	0.98822	0.824439	NA
$MOR					
	difference				
pvalue		sexM:socialPB	sexM:socialSol	sexF:socialPB	sexF:socialSol

	sexM:socialPB	NA	0.999995	-0.98402	0.330487
	sexM:socialSol	0.699819	NA	-1.98401	-0.66951
	sexF:socialPB	0.609285	0.482176	NA	1.314505
	sexF:socialSol	0.839055	0.793307	0.443256	NA
$r18S					
	difference				
pvalue		sexM:socialPB	sexM:socialSol	sexF:socialPB	sexF:socialSol
	sexM:socialPB	NA	0.498663	0.031447	1.01339
	sexM:socialSol	0.419681	NA	-0.46722	0.514727
	sexF:socialPB	0.955814	0.599333	NA	0.981943
	sexF:socialSol	0.102503	0.459069	0.119823	NA
$V1aR					
	difference				
pvalue		sexM:socialPB	sexM:socialSol	sexF:socialPB	sexF:socialSol
	sexM:socialPB	NA	1.635519	0.532984	-0.89915
	sexM:socialSol	0.434986	NA	-1.10254	-2.53467
	sexF:socialPB	0.705167	0.59971	NA	-1.43213
	sexF:socialSol	0.464576	0.20936	0.269615	NA

**POA (POA)**	post.mean	l-95%	u-95%	eff.samp	pMCMC
geneD1R	3.47028	2.55003	4.31307	1000	<0.001
geneD2R	4.34809	3.50166	5.17739	1000	<0.001
geneITR	0.08649	-0.81952	0.86331	1000	0.804
geneMOR	4.16535	3.23615	5.15936	1000	<0.001
geneV1aR	-0.35394	-1.31339	0.70585	1000	0.494
gener18S	12.62434	11.9034	13.27657	1123	<0.001
geneD1R:sexF	-0.25467	-1.51291	0.9873	1000	0.646
geneD2R:sexF	-0.14761	-1.36827	0.98772	1000	0.844
geneITR:sexF	0.27825	-0.80299	1.61599	1000	0.65
geneMOR:sexF	-0.16301	-1.5625	1.10806	990.2	0.814
geneV1aR:sexF	-0.48373	-2.08242	0.70981	1000	0.512
gener18S:sexF	0.18975	-0.48194	0.88497	1000	0.61
geneD1R:socialSol	0.61762	-1.46324	2.66489	1000	0.584
geneD2R:socialSol	-0.6874	-2.7015	1.37032	1000	0.492
geneITR:socialSol	-1.30697	-3.55063	0.88706	1000	0.244
geneMOR:socialSol	-0.68967	-2.91948	1.47857	1000	0.552
geneV1aR:socialSol	-0.8422	-2.99982	1.50492	1000	0.48
gener18S:socialSol	0.75208	-0.09433	1.62248	1000	0.108
geneD1R:sexF:socialSol	0.5488	-1.82717	2.6809	1167.5	0.652
geneD2R:sexF:socialSol	1.20831	-1.1817	3.37319	1000	0.298
geneITR:sexF:socialSol	1.50295	-0.97492	3.93551	1000	0.238
geneMOR:sexF:socialSol	1.25509	-1.24497	3.92943	1000	0.318
geneV1aR:sexF:socialSol	0.81277	-1.91626	3.16279	1000	0.556
gener18S:sexF:socialSol	0.68544	-0.20761	1.52233	1000	0.108

POA (POA) Pair-wise p values

$D1R					
	difference				
pvalue		sexM:socialPB	sexM:socialSol	sexF:socialPB	sexF:socialSol
	sexM:socialPB	NA	0.891042	-0.36742	1.315372
	sexM:socialSol	0.561675	NA	-1.25846	0.424331
	sexF:socialPB	0.686977	0.40222	NA	1.682788
	sexF:socialSol	0.112295	0.774482	0.032288	NA
$D2R					
	difference				
pvalue		sexM:socialPB	sexM:socialSol	sexF:socialPB	sexF:socialSol
	sexM:socialPB	NA	-0.99171	-0.21296	0.538564
	sexM:socialSol	0.508362	NA	0.778753	1.530273
	sexF:socialPB	0.816171	0.599704	NA	0.75152
	sexF:socialSol	0.498257	0.276759	0.35441	NA
$ITR					
	difference				
pvalue		sexM:socialPB	sexM:socialSol	sexF:socialPB	sexF:socialSol
	sexM:socialPB	NA	-1.88556	0.401424	0.684165
	sexM:socialSol	0.248864	NA	2.28698	2.56972
	sexF:socialPB	0.65417	0.157506	NA	0.282741
	sexF:socialSol	0.403625	0.100053	0.733244	NA
$MOR					
	difference				
pvalue		sexM:socialPB	sexM:socialSol	sexF:socialPB	sexF:socialSol
	sexM:socialPB	NA	-0.99498	-0.23517	0.580553
	sexM:socialSol	0.551456	NA	0.759808	1.575536
	sexF:socialPB	0.812459	0.651128	NA	0.815728
	sexF:socialSol	0.510823	0.334236	0.379342	NA
$V1aR					
	difference				
pvalue		sexM:socialPB	sexM:socialSol	sexF:socialPB	sexF:socialSol
	sexM:socialPB	NA	-1.21504	-0.69787	-0.74033
	sexM:socialSol	0.468099	NA	0.517168	0.47471
	sexF:socialPB	0.499057	0.757094	NA	-0.04246
	sexF:socialSol	0.423515	0.768798	0.963275	NA
$r18S					
	difference				
pvalue		sexM:socialPB	sexM:socialSol	sexF:socialPB	sexF:socialSol
	sexM:socialPB	NA	1.085015	0.273756	2.34765
	sexM:socialSol	0.088925	NA	-0.81126	1.262635
	sexF:socialPB	0.60507	0.347608	NA	2.073894

	sexF:socialSol	0.000266	0.084652	0.000437	NA

**Vc (Str/CP)**	post.mean	l-95%	u-95%	eff.samp	pMCMC
geneD1R	2.65814	1.79555	3.7582	1000	<0.001
geneD2R	4.01137	3.06902	4.92022	1000	<0.001
geneITR	-0.13941	-1.16918	0.85515	1000	0.78
geneMOR	3.19274	1.60444	4.75663	837.1	<0.001
geneV1aR	-2.00819	-3.30649	-0.87314	1000	<0.001
gener18S	12.22007	11.47743	13.01413	1000	<0.001
geneD1R:sexF	-0.24252	-1.72257	1.31244	1000	0.75
geneD2R:sexF	0.2277	-1.28501	1.66822	1000	0.754
geneITR:sexF	-0.7645	-2.30366	0.73032	1025.2	0.35
geneMOR:sexF	0.91649	-1.67809	3.07477	1000	0.454
geneV1aR:sexF	-0.6868	-2.71887	1.26281	990.3	0.508
gener18S:sexF	0.07285	-0.77938	0.87739	1000	0.88
geneD1R:socialSol	0.5747	-1.53936	2.64884	1000	0.598
geneD2R:socialSol	-1.29885	-3.36558	0.68517	1177.6	0.228
geneITR:socialSol	-2.76981	-5.75164	-0.04515	1000	0.052
geneMOR:socialSol	-1.27822	-4.44769	2.09977	1000	0.448
geneV1aR:socialSol	-2.51782	-6.1199	1.26103	1000	0.192
gener18S:socialSol	0.39303	-0.4916	1.22719	1000	0.386
geneD1R:sexF:socialSol	-0.64881	-3.18752	1.93847	838.6	0.612
geneD2R:sexF:socialSol	0.43111	-2.257	2.84365	1000	0.758
geneITR:sexF:socialSol	2.74546	-0.44973	6.22583	1000	0.088
geneMOR:sexF:socialSol	-0.38578	-4.4329	3.622	1000	0.812
geneV1aR:sexF:socialSol	3.20803	-1.07125	7.52059	1000	0.114
gener18S:sexF:socialSol	0.30727	-0.60411	1.24304	1000	0.52

Vc (Str/CP) Pair-wise p values

$D1R					
	difference				
pvalue		sexM:socialPB	sexM:socialSol	sexF:socialPB	sexF:socialSol
	sexM:socialPB	NA	0.82911	-0.34988	-0.4568
	sexM:socialSol	0.596079	NA	-1.17899	-1.28591
	sexF:socialPB	0.763587	0.470557	NA	-0.10692
	sexF:socialSol	0.613972	0.391513	0.917392	NA
$D2R					
	difference				
pvalue		sexM:socialPB	sexM:socialSol	sexF:socialPB	sexF:socialSol
	sexM:socialPB	NA	-1.87385	0.328499	-0.92339
	sexM:socialSol	0.217034	NA	2.202347	0.950463
	sexF:socialPB	0.764915	0.157432	NA	-1.25188
	sexF:socialSol	0.273209	0.523657	0.203337	NA
$ITR					

	difference				
pvalue		sexM:socialPB	sexM:socialSol	sexF:socialPB	sexF:socialSol
	sexM:socialPB	NA	-3.996	-1.10294	-1.13808
	sexM:socialSol	0.061854	NA	2.893057	2.857914
	sexF:socialPB	0.341241	0.180948	NA	-0.03514
	sexF:socialSol	0.240267	0.172433	0.974438	NA
$MOR					
	difference				
pvalue		sexM:socialPB	sexM:socialSol	sexF:socialPB	sexF:socialSol
	sexM:socialPB	NA	-1.84408	1.32221	-1.07844
	sexM:socialSol	0.449884	NA	3.166288	0.765641
	sexF:socialPB	0.461679	0.208495	NA	-2.40065
	sexF:socialSol	0.469333	0.750179	0.143498	NA
$V1aR					
	difference				
pvalue		sexM:socialPB	sexM:socialSol	sexF:socialPB	sexF:socialSol
	sexM:socialPB	NA	-3.63245	-0.99084	0.004915
	sexM:socialSol	0.199562	NA	2.641602	3.637362
	sexF:socialPB	0.499566	0.370046	NA	0.99576
	sexF:socialSol	0.996439	0.195251	0.452305	NA
$r18S					
	difference				
pvalue		sexM:socialPB	sexM:socialSol	sexF:socialPB	sexF:socialSol
	sexM:socialPB	NA	0.567019	0.105095	1.115413
	sexM:socialSol	0.383312	NA	-0.46192	0.548393
	sexF:socialPB	0.867387	0.623853	NA	1.010318
	sexF:socialSol	0.142133	0.478285	0.157653	NA

**TPp (VTA)**	post.mean	l-95%	u-95%	eff.samp	pMCMC
geneD1R	0.55477	-0.56094	1.76715	1231.9	0.352
geneD2R	2.26394	1.17787	3.14334	1120.8	<0.001
geneITR	-1.17488	-2.37169	-0.05856	1000	0.056
geneMOR	2.00858	1.01206	3.00135	928.6	<0.001
geneV1aR	-2.63766	-4.08429	-1.38868	1000	<0.001
gener18S	11.22024	10.45378	12.01931	1000	<0.001
geneD1R:sexF	1.22318	-0.52844	2.80597	1010	0.162
geneD2R:sexF	0.97531	-0.41269	2.35845	1000	0.172
geneITR:sexF	0.5566	-0.97961	2.17189	938.9	0.504
geneMOR:sexF	0.41159	-1.02057	1.9225	997.5	0.582

geneV1aR:sexF	0.78672	-0.95336	2.73478	1000	0.388
gener18S:sexF	0.29173	-0.51454	1.13936	1000	0.51
geneD1R:socialSol	1.50616	-0.83438	3.87766	1000	0.19
geneD2R:socialSol	-0.09204	-2.47839	2.0791	1000	0.914
geneITR:socialSol	-0.48215	-2.88487	2.41712	1146.4	0.73
geneMOR:socialSol	0.89084	-1.3751	3.12954	1000	0.44
geneV1aR:socialSol	0.29866	-2.41009	3.48911	1000	0.874
gener18S:socialSol	0.54636	-0.3464	1.3792	1000	0.244
geneD1R:sexF:socialSol	-1.68391	-4.52297	0.98605	1000	0.232
geneD2R:sexF:socialSol	-1.23208	-3.88805	1.28271	1000	0.348
geneITR:sexF:socialSol	-0.161	-3.26474	2.81771	1097	0.924
geneMOR:sexF:socialSol	-0.95217	-3.92275	1.44154	1000	0.472
geneV1aR:sexF:socialSol	-0.28906	-3.69368	3.24725	1000	0.844
gener18S:sexF:socialSol	0.20439	-0.71813	1.10849	951.6	0.678

TPp (VTA) Pair-wise p values

$D1R					
	difference				
pvalue		sexM:socialPB	sexM:socialSol	sexF:socialPB	sexF:socialSol
	sexM:socialPB	NA	2.172936	1.76467	1.508236
	sexM:socialSol	0.206875	NA	-0.40827	-0.6647
	sexF:socialPB	0.147852	0.820516	NA	-0.25643
	sexF:socialSol	0.154253	0.690657	0.807971	NA
$D2R					
	difference				
pvalue		sexM:socialPB	sexM:socialSol	sexF:socialPB	sexF:socialSol
	sexM:socialPB	NA	-0.13278	1.407081	-0.50322
	sexM:socialSol	0.937058	NA	1.539863	-0.37044
	sexF:socialPB	0.177968	0.36324	NA	-1.9103
	sexF:socialSol	0.593096	0.822446	0.052901	NA
$ITR					
	difference				
pvalue		sexM:socialPB	sexM:socialSol	sexF:socialPB	sexF:socialSol
	sexM:socialPB	NA	-0.69559	0.803004	-0.12487
	sexM:socialSol	0.721516	NA	1.498596	0.570725
	sexF:socialPB	0.493903	0.42187	NA	-0.92787
	sexF:socialSol	0.908185	0.760896	0.359986	NA
$MOR					
	difference				
pvalue		sexM:socialPB	sexM:socialSol	sexF:socialPB	sexF:socialSol

	sexM:socialPB	NA	1.285207	0.593792	0.505314
	sexM:socialSol	0.447889	NA	-0.69141	-0.77989
	sexF:socialPB	0.587829	0.683541	NA	-0.08848
	sexF:socialSol	0.598355	0.625549	0.925786	NA
$V1aR					
	difference				
pvalue		sexM:socialPB	sexM:socialSol	sexF:socialPB	sexF:socialSol
	sexM:socialPB	NA	0.430869	1.134996	1.148842
	sexM:socialSol	0.845226	NA	0.704127	0.717973
	sexF:socialPB	0.395304	0.742335	NA	0.013846
	sexF:socialSol	0.357187	0.73087	0.990855	NA
$r18S					
	difference				
pvalue		sexM:socialPB	sexM:socialSol	sexF:socialPB	sexF:socialSol
	sexM:socialPB	NA	0.788225	0.420873	1.503977
	sexM:socialSol	0.225171	NA	-0.36735	0.715752
	sexF:socialPB	0.497241	0.690728	NA	1.083104
	sexF:socialSol	0.048999	0.364969	0.114944	NA

Key: post. mean = posterior mean, l-95 % = lower 95% credible interval limit, u-95 % = upper 95% credible interval limit, pMCMC = Bayesian two-tailed p-value at alpha = 0.05. *Brain region abbreviations:* teleost: telen. = telencephalon, Dm = medial part of the dorsal telen., Vd = dorsal part of the ventral telen., Dl = lateral part of the dorsal telen., Vv/Vl = lateral and ventral part of the ventral telen., Vs = supracommissural part of the ventral telen., Vc = central part of the ventral telen., POA = pre optic area, TPp = periventricular part of the posterior tuberculum., putative mammalian homolog: blAMY = basolateral amygdala, NAcc = nucleus accumbens, HIP = hippocampus, LS = lateral septum, meAMY/BNST = medial amygdala/bed nucleus of the stria terminalis, Str = Striatum, CP = caudate putamen, VTA = ventral tegmental area.

Supplementary Table S5: Model summary of gene expression differences between male *Chaetodon* butterflyfish species within the supracommisural part of the ventral telencephalon. Results are reported as natural log-fold changes of posterior mean from the *a priori* comparison state of *Chaetodon baronessa* with a two-tailed p value.

**Vs (meAMY.BNST)**	post.mean	l-95%	u-95%	eff.samp	pMCMC
geneD1R	-3.85E+00	-9.19E+00	8.24E-03	341.655	0.02
geneD2R	1.33E+00	-7.12E-01	3.00E+00	1000	0.162
geneITR	-2.78E+00	-5.25E+00	-3.77E-01	748.042	0.006
geneMOR	3.18E+00	1.21E+00	5.18E+00	1000	0.004
geneV1aR	-2.68E+00	-4.98E+00	-5.10E-01	1000	0.008
gener18S	1.29E+01	1.20E+01	1.38E+01	1000	<0.001
geneD1R:speciesC.lun	4.75E+00	4.18E-02	1.03E+01	581.696	0.03
geneD2R:speciesC.lun	1.75E+00	-1.16E+00	4.40E+00	1000	0.204
geneITR:speciesC.lun	7.14E-01	-2.70E+00	4.16E+00	794.09	0.68
geneMOR:speciesC.lun	-4.88E-01	-4.06E+00	2.52E+00	1000	0.746
geneV1aR:speciesC.lun	2.47E+00	-4.68E-01	5.64E+00	1000	0.102
gener18S:speciesC.lun	3.61E-01	-6.57E-01	1.44E+00	1000	0.514
geneD1R:speciesC.rainf	5.44E+00	-5.06E-01	1.23E+01	621.632	0.042
geneD2R:speciesC.rainf	-8.82E-01	-4.74E+00	2.65E+00	980.066	0.654

geneITR:speciesC.rainf	-9.77E+01	-1.66E+02	1.97E+00	4.575	0.006
geneMOR:speciesC.rainf	-3.03E+00	-7.46E+00	9.41E-01	1000	0.134
geneV1aR:speciesC.rainf	-1.11E+02	-2.54E+02	-1.36E+01	3.45	<0.001
gener18S:speciesC.rainf	-1.48E-01	-1.33E+00	9.30E-01	1000	0.814
geneD1R:speciesC.trif	6.98E+00	9.17E-01	1.37E+01	491.553	0.008
geneD2R:speciesC.trif	2.38E+00	-1.36E+00	5.81E+00	1000	0.182
geneITR:speciesC.trif	-6.03E+01	-1.29E+02	-2.44E+00	5.321	0.01
geneMOR:speciesC.trif	1.76E-01	-4.20E+00	3.98E+00	1000	0.916
geneV1aR:speciesC.trif	1.29E+00	-3.28E+00	5.68E+00	1000	0.542
gener18S:speciesC.trif	9.37E-03	-1.02E+00	1.21E+00	1000	0.98
geneD1R:speciesC.vag	5.34E+00	9.25E-01	1.10E+01	421.043	0.008
geneD2R:speciesC.vag	2.63E+00	3.68E-01	5.06E+00	1000	0.032
geneITR:speciesC.vag	1.40E+00	-1.50E+00	4.58E+00	866.362	0.382
geneMOR:speciesC.vag	2.18E+00	-5.11E-01	4.90E+00	1000	0.09
geneV1aR:speciesC.vag	3.83E+00	1.17E+00	6.74E+00	1305.185	0.004
gener18S:speciesC.vag	3.65E-01	-7.42E-01	1.28E+00	1000	0.482

Vs (meAMY.BNST) Pair-wise p values

$D1R						
	difference					
pvalue		C.bar	C.lun	C.rainf	C.trif	C.vag
	C.bar	NA	6.846559	7.849495	10.06954	7.699104
	C.lun	0.072924	NA	1.002936	3.22298	0.852545
	C.rainf	0.095073	0.808614	NA	2.220044	-0.15039
	C.trif	0.033875	0.438651	0.650826	NA	-2.37043
	C.vag	0.041933	0.779403	0.968907	0.541053	NA
$D2R						
	difference					
pvalue		C.bar	C.lun	C.rainf	C.trif	C.vag
	C.bar	NA	2.528046	-1.27193	3.434479	3.791663
	C.lun	0.208957	NA	-3.79998	0.906433	1.263617
	C.rainf	0.651541	0.16869	NA	4.706412	5.063596
	C.trif	0.186504	0.741509	0.165786	NA	0.357184
	C.vag	0.030836	0.513748	0.059955	0.887718	NA
$ITR						
	difference					
pvalue		C.bar	C.lun	C.rainf	C.trif	C.vag
	C.bar	NA	1.030598	-140.947	-86.9908	2.018993
	C.lun	0.687678	NA	-141.978	-88.0214	0.988395
	C.rainf	0.047687	0.045785	NA	53.95665	142.9664
	C.trif	0.11556	0.111104	0.601884	NA	89.00975
	C.vag	0.388087	0.690695	0.044372	0.106807	NA
$MOR						
	difference					

pvalue		C.bar	C.lun	C.rainf	C.trif	C.vag
	C.bar	NA	-0.70369	-4.37093	0.25317	3.142817
	C.lun	0.767663	NA	-3.66724	0.95686	3.846507
	C.rainf	0.159047	0.265396	NA	4.624097	7.513744
	C.trif	0.933398	0.7739	0.235666	NA	2.889647
	C.vag	0.106342	0.099076	0.013392	0.338936	NA
$V1aR						
	difference					
pvalue		C.bar	C.lun	C.rainf	C.trif	C.vag
	C.bar	NA	3.559984	-160.411	1.856114	5.531158
	C.lun	0.114231	NA	-163.971	-1.70387	1.971174
	C.rainf	0.081732	0.0756	NA	162.2671	165.9422
	C.trif	0.562972	0.59314	0.078831	NA	3.675044
	C.vag	0.007693	0.327244	0.072071	0.21083	NA
$r18S						
	difference					
pvalue		C.bar	C.lun	C.rainf	C.trif	C.vag
	C.bar	NA	0.520708	-0.21374	0.013524	0.52661
	C.lun	0.508665	NA	-0.73445	-0.50718	0.005902
	C.rainf	0.796529	0.525246	NA	0.227265	0.740351
	C.trif	0.987061	0.653522	0.847257	NA	0.513086
	C.vag	0.47746	0.995432	0.499484	0.638388	NA

Key: post. mean = posterior mean, l-95 % = lower 95% credible interval limit, u-95 % = upper 95% credible interval limit, pMCMC = Bayesian two-tailed p-value at alpha = 0.05, *Brain region abbreviations:* Vs = supracommissural part of the ventral telencephalon, meAMY/BNST = medial amygdala/bed nucleus of the stria terminalis.
